# Isolation and Identification of Antibacterial Bioactive Compounds From *Bacillus megaterium* L2

**DOI:** 10.3389/fmicb.2021.645484

**Published:** 2021-03-24

**Authors:** Yudan Xie, Qiuju Peng, Yuyu Ji, Ailin Xie, Long Yang, Shuzhen Mu, Zhu Li, Tengxia He, Yang Xiao, Jinyi Zhao, Qinyu Zhang

**Affiliations:** ^1^Key Laboratory of Plant Resource Conservation and Germplasm Innovation in Mountainous Region (Ministry of Education), Collaborative Innovation Center for Mountain Ecology & Agro-Bioengineering, College of Life Sciences/Institute of Agro-bioengineering, Guizhou University, Guiyang, China; ^2^College of Life Sciences, Guizhou University, Guiyang, China; ^3^State Key Laboratory of Functions and Applications of Medicinal Plants, Guizhou Medical University, Guiyang, China; ^4^Institution of Supervision and Inspection Product Quality of Guizhou Province, Guiyang, China

**Keywords:** *Bacillus megaterium*, biocontrol, antibacterial activity, behenic acid, phenylacetic acid, β-sitosterol

## Abstract

Bacterial metabolites exhibit a variety of biologically active compounds including antibacterial and antifungal activities. It is well known that *Bacillus* is considered to be a promising source of bioactive secondary metabolites. Most plant pathogens have an incredible ability to mutate and acquire resistance, causing major economic losses in the agricultural field. Therefore, it is necessary to use the natural antibacterial compounds in microbes to control plant pathogens. This study was conducted to investigate the bio-active compounds of *Bacillus megaterium* L2. According to the activity guidance of *Agrobacterium tumefaciens* T-37, *Erwinia carotovora* EC-1 and *Ralstonia solanacearum* RS-2, five monomeric compounds, including erucamide (**1**), behenic acid (**2**), palmitic acid (**3**), phenylacetic acid (**4**), and β-sitosterol (**5**), were fractionated and purified from the crude ethyl acetate extract of *B. megaterium*. To our knowledge, all compounds were isolated from the bacterium for the first time. To understand the antimicrobial activity of these compounds, and their minimum inhibitory concentrations (MICs) (range: 0.98∼500 μg/mL) were determined by the broth microdilution method. For the three tested pathogens, palmitic acid exhibited almost no antibacterial activity (>500 μg/mL), while erucamide had moderate antibacterial activity (MIC = 500 μg/mL). Behenic acid showed MICs of 250 μg/mL against T-37 and RS-2 strains with an antibacterial activity. β-sitosterol showed significant antimicrobial activity against RS-2. β-sitosterol showed remarkable antimicrobial activity against RS-2 with an MIC of 15.6 μg/mL. In addition, with the antimicrobial activity, against T-37 (62.5 μg/mL) and against EC-1 (125 μg/mL) and RS-2 (15.6 μg/mL) strains notably, phenylacetic acid may be interesting for the prevention and control of phytopathogenic bacteria. Our findings suggest that isolated compounds such as behenic acid, β-sitosterol, and phenylacetic acid may be promising candidates for natural antimicrobial agents.

## Introduction

Increasing use of chemical pesticides to combat a variety of plant diseases has resulted in heavy soil pollution in recent years (Munoz-Leoz et al., 2013). Chemical pesticides negatively affect microbial activities and other non-target essential soil organisms such as soil microbes (Gupta et al., 2013; Sun et al., 2020) and nematodes (Zhang et al., 2019). Moreover, uncontrolled use of pesticides such as fungicides has developed resistance to pathogens; thus, making disease control difficult to achieve (Fernández-Ortuño et al., 2015; Yang et al., 2019). Biological control agents can act as a pathogen-specific, safe, and pollution-free alternative to chemicals that have negative effects on the environment and animal and human health (Goulson, 2014; Nicolopoulou-Stamati et al., 2016).

Natural resources such as microorganisms, plants, and animals are used to extract novel compounds, of which microbes are a major source for finding new antimicrobial agents. Among the microbes, *Bacillus megaterium* is a rich source of metabolites, which is well-known to produce metabolites with antibacterial and antifungal activities (Al-Thubiani et al., 2018; Mannaa and Kim, 2018). Before the widespread prevalence of *Bacillus subtilis*, *B. megaterium* had been widely used in biochemical research due to its extensive metabolic capacity and physical properties conducive to biotechnology applications (Hitchins et al., 1968; Elmerich and Aubert, 1971). The strain is a commercially available, nonpathogenic host, which has been used to produce various enzymes, such as penicillin amidase, amylase, amino acid dehydrogenase, and glucose dehydrogenase, as well as to produce recombinant proteins (Lammers et al., 2004; Malten et al., 2005; Biedendieck et al., 2011; Grage et al., 2017; Guo et al., 2017). Moreover, *B. megaterium* can synthesize vitamin B_12_ through an oxygen-independent adenosylcobalamin pathway (Eppinger et al., 2011).

*Bacillus megaterium* is an aerobic, spore-forming, and Gram-positive bacterium, widely distributed in habitats such as soil, seawater, sediments, rice paddies, dried food, honey, and milk (Lee et al., 2016). It is used in various fields because of its advantages, such as environmentally beneficial, non-pathogenic to humans and animals, small generation time, simple nutritional requirements, and strong stress resistance (Nascimento et al., 2019), especially agriculture. *B. megaterium* has been extensively studied as the bio-fungicide, biofertilizer, plant growth enhancer, plant growth-promoting rhizobacteria (PGPR) and broad-spectrum biocontrol agent in the agriculture industry (Chakraborty et al., 2006; Boostani et al., 2014; Kamal et al., 2021), which may improve the soil microbial ecosystem and reduce the number of soil-borne plant pathogens. In addition, isolated active antibacterial substances could be applied onto the soil to improve the growth of plants and reduce the number of plant pathogens (Ryan et al., 2009; Hu et al., 2013). The antibacterial activities have been extensively investigated with crude or purified extracts from various sources, but there are few studies on the isolation of antimicrobial compounds from *B. megaterium* and their antibacterial mechanism.

*Agrobacterium tumefaciens* is a Gram-negative plant pathogen causing the widespread disease known as crown gall (Aysan and Sahin, 2003). The organism can infect 93 families, 331 genera, and 643 species of plants, most of which are dicotyledonous plants; a few are gymnosperms and monocotyledonous plants (Conner and Dommisse., 1992). *Erwinia carotovora* is a widespread Gram-negative plant pathogen that causes soft rot disease in many plants and vegetables, such as potato, green peppers, celery, carrot, cabbage, and tomato (Toth et al., 2003; Rahman et al., 2012). *Ralstonia solanacearum*, formerly known as *Pseudomonas solanacearum*, is a Gram-negative bacterium and the causative agent of bacterial wilt worldwide. It can infect more than 250 plant species (Elphinstone, 2005), including potato, tomato, eggplant, pepper, ginger, banana, and tobacco. The diseases caused by these three plant pathogens lead to huge economic loss worldwide and are thus considered a major threat to agriculture. In our previous studies, *B. megaterium* L2 was identified as a potential source of antibacterial bioactive compounds. The eluted components of crude extract from *B. megaterium* L2 were found to exhibit good inhibitory activity against three common plant pathogens: *A. tumefaciens, E. carotovora*, and *R. solanacearum* (Ji et al., 2019; Zhao et al., 2019). In this study, we aimed to isolate and characterize antibacterial bioactive compounds from *B. megaterium* against plant pathogens, including *A. tumefaciens* T-37, *E. carotovora* EC-1, and *R. solanacearum* RS-2, which may help understand the mechanism of antimicrobial activity and develop novel microbial-derived pesticides in agriculture.

## Materials and Methods

### Strains

*Bacillus megaterium* L2 was isolated, screened, and identified at the Fungal Resources Laboratory of the College of Life Sciences, Guizhou University, and preserved in the China Center for Type Culture Collection (CCTCC, NO. M2012381).

*Agrobacterium tumefaciens* T-37 was purchased from the Institute of Soil Fertilizer, Chinese Academy of Agricultural Sciences, and preserved in the Fungal Resources Laboratory of the College of Life Sciences, Guizhou University.

*Erwinia carotovora* EC-1 and *R. solanacearum* RS-2 were isolated, screened, identified, and preserved at the Laboratory of Institute of Fungal Resources, Guizhou University.

### Culture Media

Beef extract peptone liquid medium was prepared using 10 g/L of peptone, 5 g/L of NaCl, 3 g/L of beef extract, and 1 L of distilled water at pH 7.4–7.6, followed by sterilization at 121°C for 20 min. The medium was used to culture three tested plant pathogens (T-37, EC-1, and RS-2 strains) to guide the fractionation of active fractions and investigate the antimicrobial activity of the compounds.

Beef extract peptone solid medium was prepared using 15–20 g/L of agar and beef paste peptone liquid medium. The three tested plant pathogens were cultured on the medium at 30°C for 24 h to obtain active bacteria.

Nutrient broth (NB) medium was prepared using 10 g/L of peptone, 5 g/L of glucose, 5 g/L of NaCl, 3 g/L of beef paste, and 1 L of distilled water at pH 7.0–7.2, followed by sterilization at 121°C for 20 min. The medium was used for the culture and fermentation of L2 strain.

Nutrient agar (NA) medium was prepared by adding 15–20 g/L of agar to NB.

### Strain Activation and Fermentation

L2 strain was cultured on NA at 30°C for 48 h to obtain active bacteria. After subculturing for 2–3 times, colonies were inoculated into NB medium and incubated at 30°C and 150 r/min for 20 h for the preparation of seed broth. Fermentation was performed according to the optimal fermentation condition for the strain L2 (Ji et al., 2018). Seed broth was transferred into a fermentation tank (400 L) containing 80 L of NB medium for large-scale cultivation and incubated with a ventilation rate of 1.5 L/min, 150 r/min at 30°C for 48 h.

### Extract Preparation

The bacterial cells obtained after fermentation were spray-dried to form a pulverized powder (35 kg). Of the powder, 5 kg was reflux extracted thrice with 95% ethanol (each 15 L) at 80°C for 4 h. Then the total ethanol extract was collected by filtration using Buchner funnel and concentrated by rotary evaporator at 40°C to obtain the crude ethanol extract. A total of 100 g of the crude extract was suspended in water (200 mL) followed by subsequent fractionation using petroleum ether, ethyl acetate, n-butanol, and water (200 mL for each solvent). The four organic fractions were concentrated and dried under reduced pressure by rotary evaporator at 40°C, respectively, and then subject to the determination of antibacterial activity.

### Antibacterial Activity

We determined the antibacterial activity of the four organic extracts and active fractions against T-37, EC-1, and RS-2 strains used as indicator bacteria. Each fraction of the organic extracts and active fractions were dissolved in dimethyl sulfoxide (DMSO) respectively to obtain a solution with a concentration of 100 mg/mL. Ten microliter of the solutions were added to 1 mL of beef extract peptone liquid medium to final concentration of 1 mg/mL, and 10% each bacterial suspension (10^8^ CFU/mL) was inoculated and incubated at 37°C and 120 r/min for 12 h. Chloramphenicol (CHL) was used as the positive control, and DMSO was used as the solvent control. All tests were performed in triplicate. Cell density (OD_*max*_) was measured using a UV-Vis Spectrophotometer. The inhibition rate was calculated using the following equation:

$$I_{R}=\frac{{\text{OD}}_{0}-{\text{OD}}_{1}}{{\text{OD}}_{0}}\times 100\%$$

where I_*R*_ represents the bacterial inhibition rate, OD_1_ represents the OD_*max*_ of the experimental group, and OD_0_ represents the OD_*max*_ of the solvent control group. The OD_*max*_ for T-37, EC-1, and RS-2 were 400, 490, and 420 nm, respectively.

### Isolation and Purification of the Ethyl Acetate Phase

The ethyl acetate extract (814.8 g) was separated from the combined organic extract by silica gel column chromatography. For gradient elution, different solvent systems were used, such as pure petroleum ether, petroleum ether-ethyl acetate solution (petroleum ether:ethyl acetate = 100:1, 50:1, 20:1, 10:1, 5:1, 2:1, 1:1, v/v), ethyl acetate-methanol solution (ethyl acetate:methanol = 30:1, 20:1, 10:1, 5:1, 2:1, 1:1, v/v), and pure methanol. Fractions (200 mL) were collected, concentrated, and merged with the fractions having the same polarity composition as determined by TLC analysis. Finally, ten fractions were obtained: B1–B10. The antimicrobial activity-guided fractionation of bioactive fractions B2, B3, and B4 was performed as follows:

B2 (13.116 g) fraction was further subject to fractionation by using Sephadex LH-20 column and chloroform:methanol (1:1, v/v) as eluent. Six sub-fractions such as B2-(1–6) were obtained. Sub-fraction B2–1 (3.582 g) was further subject to column chromatography using an atmospheric pressure silica gel (200–300 mesh) and eluted using a gradient of petroleum ether, petroleum ether:ethyl acetate (40:1, v/v), and pure methanol to obtain four fractions such as B2–1-(1–4). B2–1–4 (314 mg) was further purified by column chromatography using a decompression silica gel (Silica gel H) and petroleum ether:chloroform (2:1, v/v) as eluent to get three fractions such as B2–1–4-(1–3). Fraction B2–1–4–2 showed a single spot on a TLC plate and compound **1** (28 mg) was obtained (Supplementary Figure 1).

B3 (19.566 g) fraction was first separated by atmospheric pressure column chromatography using silica gel (200–300 mesh) and petroleum ether:ethyl acetate (40:1, 20:1, 10:1, v/v) and pure methanol as eluent to get seven sub-fractions such as B3-(1–7). B3–5 (3.8 g) was rechromatographed according to the antimicrobial activity-guided fractionation using Sephadex LH-20 and chloroform:methanol (1:1, v/v) as eluent. Five fractions such as B3–5-(1–5) were obtained. Among them, B3–5–3 and B3–5–4 fractions showed strong antibacterial activity. B3–5–3 (1.249 g) was subsequently purified by using another silica gel column and petroleum ether:ethyl acetate (10:1, 2:1, v/v) and pure methanol as eluent to obtain four fractions such as B3–5–3-(1–4). Compound **2** (96 mg) was obtained from B3–5–3–3 by performing TLC analysis. Four fractions such as B3–5–4-(1–4) were obtained from B3–5–4 (0.813 g) by using a decompression silica gel chromatography column (200–300 mesh). Monomeric compound **3** (108 mg) was obtained from B3–5–4–2 (Supplementary Figure 2).

B4 (140.3 g) fraction was separated by atmospheric pressure silica gel column chromatography (200–300 mesh) using petroleum ether, petroleum ether:ethyl acetate (30:1, 10:1, v/v), and pure methanol as eluent to obtain four fractions such as B4-(1–4). Sub-fraction B4–2 (54.21 g) was fractionated by using Sephadex LH-20 column to obtain three fractions such as B4–2-(1–5). Finally, monomeric compound **4** (305 mg) was obtained by recrystallization from B4–2–3 (2.638 g) and B4–2–4 (1.538 g). Another sub-fraction B4–3 (23.33 g) was rechromatographed by column chromatography using an atmospheric pressure silica gel (200–300 mesh) and gradient elution was performed by using petroleum ether, petroleum ether:ethyl acetate (5:1, 2:1, v/v), and pure methanol to obtain four fractions such as B4–3-(1–4). Then B4–3–2 (3.547 g) was further purified by Sephadex LH-20 column chromatography using chloroform:methanol (1:1, v/v) as eluent to obtain seven fractions such as B4–3–2-(1–7). Monomeric compound **5** (367 mg) was obtained from B4–3–2–3 (Supplementary Figure 3).

### Identification of Compounds

The monomeric compounds were identified by structural characterization using UV spectrometry, electron spray ionization mass spectrometry (ESI-MS), electron ionization mass spectrometry (EI-MS), nuclear magnetic resonance (NMR) spectroscopy. Chloroform was used as a solvent. The obtained spectra were compared with the spectral data values of known compounds in the literature to determine the structures of the compounds.

### Determination of the Minimum Inhibitory Concentration

The minimal inhibitory concentrations were measured by broth microdilution method (Kayser and Kolodziej, 1997). Minimum inhibitory concentration (MIC) measurement was conducted to evaluate the antibacterial activity of compounds **1–5** against the three Gram-negative bacteria (T-37, EC-1, and RS-2 strains). The compounds were dissolved in DMSO (1 mg/mL) and diluted at concentrations of (500, 250, 125, 62.5, 31.3, 15.6, 7.81, 3.91, 1.95, 0.98 μg/mL) by twofold dilutions on a 96-well plate. The bacterial suspension was adjusted to a density of bacterial cells of 10^6^ CFU/mL. To each well, 100 μL of each bacterial suspension was inoculated and incubated at 37°C for 24 h. At the end of the incubation period, the MICs values were recorded as the lowest concentrations of the substance that had no visible bacterial turbidity. Tests using DMSO as solvent control and Chloramphenicol as positive control were carried out in parallel. All tests were performed in triplicate.

### Statistical Analysis

All experiments were performed in triplicate and expressed as mean ± standard deviation (SD). One-way analysis of variance (ANOVA) was performed for data analysis using IBM SPSS version 21.0. The differences among groups were evaluated by performing Duncan’s test, and a *p*-value of <0.05 was considered significant.

## Results

### Antibacterial Activities of Organic Extracts

**FIGURE 1:**
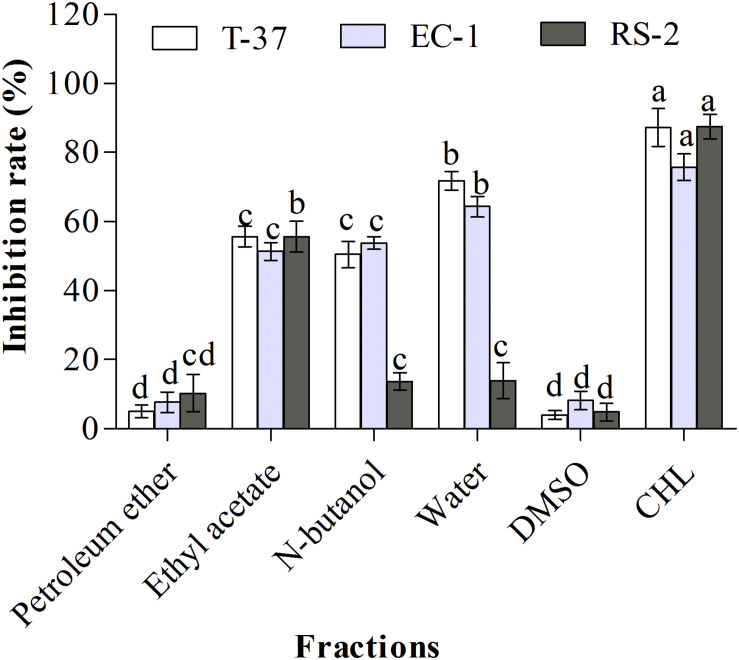
Antibacterial activities of organic extract fractions against the test bacteria. T-37, EC-1, and RS-2 represent *Agrobacterium tumefaciens* T-37, *Erwinia carotovora* EC-1, and *Ralstonia solanacearum* RS-2, respectively. DMSO represents dimethyl sulfoxide. CHL represents chloramphenicol. Different lowercase letters indicate significant differences (*P* < 0.05).

The chemical composition of natural products is complex and variable. Normally, strong lipophile compounds are eluted using petroleum ether (low polarity), then the intermediate lipophile compounds using ethyl acetate and n-butanol (medium polarity), and highly polar compounds using water (high polarity). The antibacterial effects of L2 crude extract were different for the different solvents used for elution (Figure 1). Compared with the solvent control, ethyl acetate, n-butanol, and water fractions of L2 crude extract showed significant inhibitory effects against the tested bacteria (*P* < 0.05), whereas no significant difference was observed between the petroleum ether fraction and the solvent control. The aqueous phase of L2 crude extract showed the highest inhibition rate against T-37 and EC-1 (*P* < 0.05, 71.8 and 64.37%, respectively). The inhibition rate of the n-butanol phase was more than 50% against T-37 and EC-1. However, the inhibition rates of aqueous and n-butanol phases (13.90 and 13.69%, respectively) were lower than that of ethyl acetate phase (55.67%) against RS-2. Only the ethyl acetate phase exhibited an inhibitory effect against all the tested bacteria, and the rate was more than 50%. The petroleum ether phase did not show an inhibitory effect against the test bacteria. The antibacterial bioactive compounds were present in polar and highly polar fractions, whereas the compounds were absent in the less polar fraction.

### Isolation and Purification of Ethyl Acetate Extract

**FIGURE 2:**
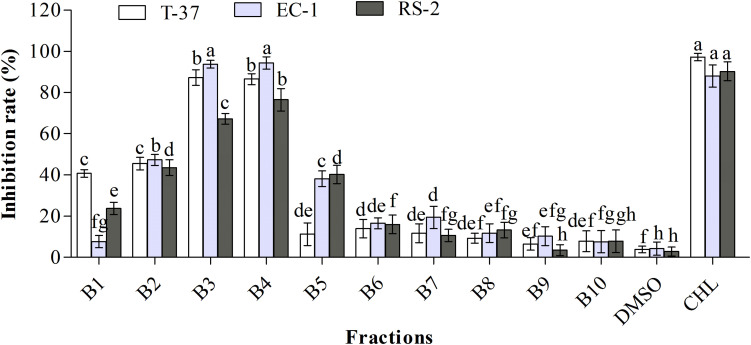
Antibacterial activities of the sub-fractions of the ethyl acetate extract against the test bacteria. T-37, EC-1, and RS-2 represent *Agrobacterium tumefaciens* T-37, *Erwinia carotovora* EC-1, and *Ralstonia solanacearum* RS-2, respectively. DMSO represents dimethyl sulfoxide. CHL represents chloramphenicol. Different lowercase letters indicate significant differences (*P* < 0.05).

Ten fractions obtained from the ethyl acetate extract were screened to determine the distribution of bioactive compounds (Figure 2). The results clearly showed that three sub-fractions B2, B3, and B4 exhibited strong antibacterial activities against the test bacteria. Specifically, B1–B4 fractions showed stronger antibacterial activities than other fractions against T-37, and their inhibition rates were 40.73, 45.52, 87.24, and 86.52%, respectively, suggestive of the high probability of bioactive compounds in B3 and B4 fractions. Moreover, the antibacterial bioactive compounds against EC-1 and RS-2 were present in a high amount in B2–B5 fractions, especially in B3 and B4, with the inhibition rates of 93.79, 94.37, and 67.24, 76.52%, respectively. The antibacterial activities of B3 and B4 fractions against T-37, RS-2, and EC-1 were significantly higher than the other fractions, with no significant differences against T-37 and EC-1, but significant difference against RS-2 (*P* < 0.05). The antibacterial activity of B2 fraction against the test bacteria was around 45%, which is more than the remaining fractions. Therefore, antibacterial activity guided fractionation of B2, B3, and B4 sub-fractions was performed for further separation and purification. Finally, compound **1** was obtained from the B1 fraction, compounds **2** and **3** from the B3 fraction, and compounds **4** and **5** from the B4 fraction.

### Identification of Compounds

**FIGURE 3:**
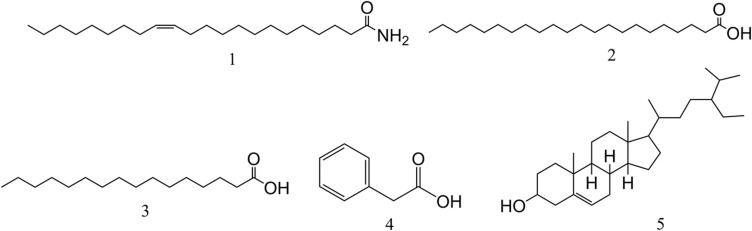
The chemical structures of compounds isolated from L2. Compounds 1∼5 are erucamide, behenic acid, palmitic acid, phenylacetic acid, and β-sitosterol, respectively.

The chemical structures of all compounds were determined by MS and NMR spectroscopy (^1^H and ^13^C) and compared with the spectral data values of known compounds previously reported in the literature. Five monomeric compounds were identified as erucamide (**1**), behenic acid (**2**), palmitic acid (**3**), phenylacetic acid (**4**), and β-sitosterol (**5**) (Figure 3).

Compound **1** (28 mg): CH_3_(CH_2_)_7_CH = CH(CH_2_)_11_CONH_2_, white powder. EI-MS (*m/z*): 337 [M]^+^; ^13^C-NMR (101 MHz, CDCl_3_) δ: 176.2 (C=O), 129.9 (C=C), 129.8 (C=C), 36.0, 31.9, 29.8, 29.6, 29.6, 29.6, 29.5, 29.5, 29.5, 29.4, 29.4, 29.4, 29.3, 29.2, 27.3, 27.2, 25.6, 22.7, 14.1. Comparing with the spectral data in the literature (Cravatt et al., 1995), compound **1** was identified as erucamide (Supplementary Figure 4).

Compound **2** (96 mg): C_22_H_44_O_2_, white powder. ESI-MS (*m/z*): 339 [M+H]^+^; ^1^H-NMR (600 MHz, CDCl_3_) *δ*: 2.36 (2H, t, *J* = 7.2 Hz, H-2), 1.65 (2H, p, *J* = 7.2 Hz, H-3), 1.27 (36H, br.s, H-4∼21), 0.89 (3H, t, *J* = 7.2 Hz, H-22); ^13^C-NMR (151 MHz, CDCl_3_) δ: 34.0 (C-2), 22.7∼29.7 (C-3∼19, 21), 32.0 (C-20), 14.1(C-22). Comparing with the spectral data in the literature (Ding et al., 2014), compound **2** was identified as behenic acid (Supplementary Figures 5, 6).

Compound **3** (108 mg): C_16_H_32_O_2_, white scaly crystal. Its spectral data belonged to the following: ESI-MS (*m/z*): 256 [M-H]^–^, ^1^H-NMR (600 MHz, CDCl_3_) δ: 2.34 (2H, t, *J* = 7.5 Hz), 1.63 (2H, m, *J* = 15.0, 7.4 Hz), 1.30∼1.25 (24H, br.s,12 × -CH2-), 0.88 (3H, t, *J* = 7.0Hz); ^13^C-NMR (101 MHz, CDCl_3_) δ 179.93 (C-1), 34.02 (C-2), 24.68 (C-3), 29.06 (C-4, 13), 29.24 (C-5, 12), 29.59 (C-6, 7, 8, 9, 10, 11), 31.93 (C-14), 22.69 (C-15), 14.10 (C-16) (Supplementary Figures 7, 8). Compound **3** was identified as palmitic acid by comparing with the spectral data in the literature (Wu et al., 1997).

Compound **4** (305 mg): C_8_H_8_O_2_, white crystal. EI-MS (*m/z*): 136 [M]^+^, 117, 107, 98, 91, 77, 65, 51, 39, 31. ^1^H-NMR (600 MHz, CDCl_3_) δ: 7.26 (5H, m, Ar-H), 3.64 (2H, s, CH_2_). By comparing with the spectral data in the literature (Kim et al., 2018), compound **4** was identified as phenylacetic acid (Supplementary Figure 9).

Compound **5** (367 mg): C_29_H_50_O, white needle-shaped crystal. EI-MS (*m/z*): 414 [M]^+^, ^1^H-NMR (500 MHz, CDCl_3_) *δ*: 5.30 (1H, br.d, H-6), 3.65 (1H, m, H-3), 0.96 (3H, s, H-19), 0.88 (3H, d, *J* = 6.6 Hz, H-21), 0.82 (3H, t, *J* = 7.5 Hz, H-29), 0.80 (3H, d, *J* = 6.8 Hz, H-27), 0.77 (3H, d, *J* = 6.8 Hz, H-26), 0.64 (3H, s, H-18) (Figure 3); ^13^C-NMR (125 MHz, CDCl_3_) *δ*: 37.2 (C-1), 31.4 (C-2), 71.6 (C-3), 42.1 (C-4), 140.8 (C-5), 121.6 (C-6), 31.8 (C-7), 31.8 (C-8), 50.0 (C-9), 36.4 (C-10), 21.0 (C-11), 39.7 (C-12), 42.2 (C-13), 56.7 (C-14), 24.2 (C-15), 28.2 (C-16), 55.9 (C-17), 11.8 (C-18), 19.3 (C-19), 36.1 (C-20), 18.9 (C-21), 33.8 (C-22), 29.0 (C-23), 45.7 (C-24), 25.9 (C-25), 18.7 (C-26), 19.7 (C-27), 23.0 (C-28), 11.8 (C-29) (Supplementary Figures 10, 11). Compound **5** was identified as β-sitosterol by comparing with the spectral data in the literature (Li et al., 2008).

### Antibacterial Activities of Monomeric Compounds

**FIGURE 4:**
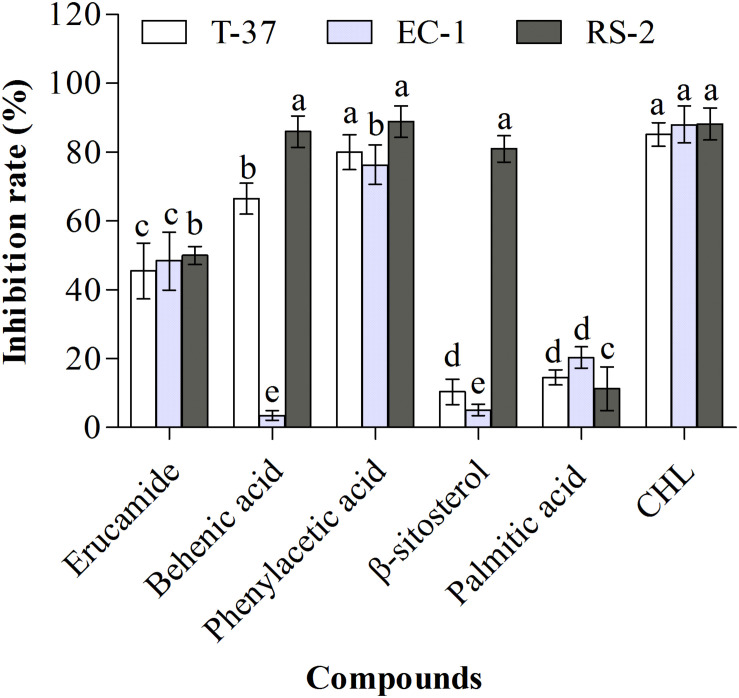
Antibacterial activities of the monomeric compounds against the test bacteria. T-37, EC-1, and RS-2 represent *Agrobacterium tumefaciens* T-37, *Erwinia carotovora* EC-1, and *Ralstonia solanacearum* RS-2, respectively. CHL represents chloramphenicol. Different lowercase letters indicate significant differences (*P* < 0.05).

The antibacterial activities of monomeric compounds were determined against the strains T-37, RS-2, and EC-1, and the results showed that not all compounds exhibit antibacterial activity (Figure 4). The antibacterial activities of the five monomeric compounds against T-37 were in the following order: phenylacetic acid > behenic acid > erucamide > palmitic acid > β-sitosterol. In addition, their antibacterial activities against EC-1 and RS-2 were in the following order: phenylacetic acid > erucamide > palmitic acid > β-sitosterol > behenic acid and phenylacetic acid > behenic acid > β-sitosterol > erucamide > palmitic acid, respectively. The test bacteria were extremely sensitive to phenylacetic acid and the inhibition rates were more than 75% in contrast to palmitic acid. Furthermore, behenic acid exhibited a strong antibacterial effect against T-37 (66.55%) and RS-2 (86.03%) but distinct inhibition was not observed against EC-1 (3.57%), indicating that behenic acid has an inhibitory effect against some bacteria. β-Sitosterol exhibited the strongest antibacterial activity only against RS-2 (81.01%), whereas against T-37 and EC-1 were 10% and 20%, respectively. Furthermore, erucamide showed 50% inhibition against the tested bacteria, its antibacterial ability was not given priority.

### Determination of the MIC

The compounds were tested against the three Gram-negative bacteria for growth inhibition, using serial dilutions of each compound with a maximum concentration of 500 μg/mL to minimize solubility problems during the assay. In this study, CHL was used as a positive control. As displayed in Table 1, compared with other compounds, phenylacetic acid exhibited a stronger antibacterial effect against the three tested bacteria, especially RS-2 (MIC = 15.6 μg/mL), followed by erucic amide, whose MIC was 500 μg/mL, while palmitic acid showed no inhibitory effect, with MIC up to 500 μg/mL. In addition, behenic acid exhibited almost full inhibition of bacterial growth against T-37 and RS-2 at 250 μg/mL. Although β-sitosterol displayed a remarkable antimicrobial activity against RS-2 indicated by an MIC value of 31.3 μg/mL, the MIC values for the other two strains were over 500 μg/mL. In summary, the compounds tested were generally found to possess only weak to moderate antimicrobial, but phenylacetic acid and β-sitosterol have strong antibacterial effects.

**TABLE 1:** The minimum inhibitory concentration (μg/mL) of the compounds.

Compounds	T-37	EC-1	RS-2
Erucamide (**1**)	500	500	500
Behenic acid (**2**)	250	>500	250
Palmitic acid (**3**)	>500	>500	>500
Phenylacetic acid (**4**)	62.5	125	15.6
β-sitosterol (**5**)	>500	>500	31.3
CHL	3.91	15.6	15.6

## Discussion

In this study, the three Gram-negative plant pathogens T-37, EC-1, and RS-2 were used for the screening of antimicrobial compounds extracted from *B. megaterium*. The bioactive compound present in the aqueous phase of crude extract exhibited a strong antibacterial activity against strains EC-1 and T-37 but its high polarity and boiling point was inconvenient for vacuum concentration and solvent recovery. Bioactive compounds were not isolated and purified from the petroleum ether fraction because of its low polarity. Furthermore, the ethyl acetate fraction exhibited high antibacterial activity against the three test bacteria compared with the n-butanol fraction, and the active compound isolated from this fraction exhibited a medium polarity. Therefore, we used ethyl acetate as the organic extraction solvent for the isolation of bioactive compounds from strain L2 in the subsequent steps. To select the fraction exhibiting the highest inhibition, we performed the antibacterial activity assay after each separation step.

*Bacillus* spp., including *B. megaterium*, have gained attention as biocontrol agents because of antagonistic or antibacterial activity against phytopathogens (Quigley, 2010; Fira et al., 2018). The underlying mechanism of the antibacterial activity of *Bacillus* is the production of inhibitory metabolites. For instance, surfactin isolated from *B. subtilis* demonstrated inhibitory effects against plant pathogenic fungi such as *Botrytis cinerea*, *Sclerotinia sclerotiorum*, *Colletotrichum gloeosporioides* (Płaza et al., 2013). 2,5-Diketopiperazines isolated from *Bacillus* sp. N strain also showed high antimicrobial activity against plant pathogenic fungi (Nishanth Kumar et al., 2012). Iturin A2, an anti-*Rhizoctonia solani* peptide, was isolated from *B. megaterium* B196 (Qin, 2013). The active monomeric compound 12-hydroxyjasmonic acid isolated from *B. megaterium* LB01 exhibited an inhibitory effect against *C. gloeosporioides* (Ding et al., 2020). Erucamide, behenic acid, palmitic acid, phenylacetic acid, and β-sitosterol isolated from *B. megaterium* L2 showed definite antibacterial activities on the tested bacteria except palmitic acid in the present study. Among them, phenylacetic acid exhibited high antibacterial activity against all the tested bacteria, showing the active compounds with antibacterial potential were successfully isolated from the metabolites of *B. megaterium* L2.

Five monomeric compounds were isolated from the ethyl acetate phase of *B. megaterium* L2, and identified as erucamide, behenic acid, palmitic acid, phenylacetic acid, and β-sitosterol. We believe that these compounds have been isolated from *B. megaterium* for the first time. Among them, erucamide, also known as cis-13-docosenoic acid, is a bioactive fatty acid amide, which has been isolated from plants (Kim et al., 2018), animals (Hamberger and Stenhagen, 2003), and microorganisms (Tamilmani et al., 2018). Although its exact biological activity remains unclear, it has been found to cause angiogenesis and angiogenic activity (Wakamatsu et al., 1990), exhibit antidepressant and anxiolytic in mice (Li et al., 2017) and stimulate nitrate reductase and nitrite reductase (Lu et al., 2014; Sun et al., 2016). In the present study, erucamide showed 50% inhibition against all the test bacteria (MIC = 500 μg/mL). However, in another study, erucamide isolated from *Trichoderma longibrachiatum* showed almost no antibacterial activity against all the tested pathogens (Zhang, 2015), suggesting that erucamide had different antimicrobial activity against different pathogens, but was not the best choice of antibacterial agent for tested bacteria.

Besides plants, phenylacetic acid can also be produced by many microorganisms, including *Bacillus* (Kawazu et al., 1996), *Enterobacter cloacae* (Slininger et al., 2004), *R. solani* (Bartz et al., 2013), *Burkholderia cepacia* (Sopheareth et al., 2013), and *Bacillus fortis* (Akram et al., 2016). Phenylacetic acid is considered a natural auxin in plants (Wightman and Lighty, 1982), could improve bud elongation and regeneration efficiency of plants such as chili pepper (Husain et al., 1999) and sunflower (Dhaka and Kothari, 2002) *in vitro*. Some microorganisms can utilize phenylacetic acid during their metabolic process, such as *Penicillium chrysogenum* uses phenylacetic acid as a precursor of penicillin G (Hillenga et al., 1995) and *R. solanacearum* uses phenylalanine and phenylacetic acid as the sole carbon and nitrogen source (Akram et al., 2016). Moreover, phenylacetic acid exhibited strong antibacterial and antifungal activities against a wide range of plant pathogens, as reported in previous studies (Hwang et al., 2001; Kim et al., 2004). As expected, our results showed that the tested bacteria were most sensitive to phenylacetic acid, and T-37, EC-1, and RS-2 strains were completely inhibited by 62.5, 125, 15.6 μg/mL, respectively, indicating that phenylacetic acid had direct antibacterial activity against phytopathogens. So, the study suggested that the presence of phenylacetic acid ethyl acetate extract of L2 strain might contribute to its potency of growth inhibition against tested bacteria.

Both behenic acid and palmitic acid are saturated fatty acids (SFA) widely found in plants that have been shown to raise cholesterol in humans (Zock et al., 1994; Cater and Denke, 2001). Among them, it was confirmed that lipid synthesis involved in the mTOR/S6K1/SREBP-1c pathways are mainly related to palmitic acid in HepG2 cells (Zhou et al., 2018). Many studies have demonstrated that palmitic acid is the main component of a variety of extracts, but most of them only study the antibacterial activity of the extracts, and few studies on the antibacterial activity of its monomer compounds. For example, palmitic acid has been shown to have no antibacterial activity against bacteria in a previous report (Li et al., 2001), but was the main antibacterial compound in *Kigelia africana* (Grace et al., 2002) and *Pentanisia prunelloides* (Yff et al., 2002). We found that no antibacterial activity was exhibited by palmitic acid (MIC > 500 μg/mL), suggesting that the compound is not considered as an antimicrobial agent for the tested plant pathogenic bacteria. On the other hand, behenic acid is also called docosanoic acid, which is isolated from the ethyl acetate fraction of *Teucrium labiosum* exhibited inhibitory activity against *Septoria zeicola* (Pi, 2009). The inhibitory effect of enrofloxacin-containing docosanoic acid solid lipid nanoparticles displayed 2.5–10 times against *Salmonella* CVCC541 than free enrofloxacin at three concentrations of 0.06, 0.24, and 0.6 μg/mL (Xie et al., 2017). We also found a significant antibacterial effect of behenic acid against the strains T-37 and RS-2 at 250 μg/mL, which indicated that behenic acid has moderate antibacterial activity, may be the main antimicrobial compound of L2 strain and could be applied for biological control. To enhance its antibacterial activity, a series of derivatives can be synthesized.

β-sitosterol is a safe, nontoxic, effective natural micronutrient, which is found in all oil producing plants, fruit, vegetables, grains, seeds, and trees (Sen et al., 2012). β-sitosterol is also a highly active compound and has been reported to have amazing potential health benefits in medicine and food, including anti-inflammatory, antipyretic, and anti-thrombotic activities (Gupta et al., 1980; Gogoi et al., 2018). In addition, β-sitosterol has an antibacterial activity. Experimental studies have shown that β-sitosterol has antibacterial activity against *Staphylococcus aureus* and *Escherichia coli.* According to Sen et al. (2012) and Joy Hoskeri et al. (2012) studies, β-sitosterol inhibited the growth of *S. aureus* and *E. coli* by (17.83 ± 0.58 and 14.5 ± 1.84 mm) and (13 and 14 mm), respectively. Our results showed that the yield of β-sitosterol was the highest among all isolated compounds (367 mg), and the antibacterial activity of β-sitosterol against RS-2 (81.01%, MIC = 31.3 μg/mL) was higher than that of T-37 and EC-1 strains. In conclusion, β-sitosterol is also the main antimicrobial compound of L2 strain, and can be used as an antimicrobial agent for agricultural plant pathogens, especially *R. solanacearum*. To increase the yield of β-sitosterol, optimization of fermentation conditions or mutagenesis of a high-yield strain can be employed.

## Conclusion

Nowadays, chemical and physical agents are used to control plant pathogens but pose a potential risk to the environment and animal and human health. The ability of microorganisms to control plant pathogens is mainly because of the presence of antimicrobial bioactive compounds. Therefore, isolation and identification of bioactive compounds are essential for the development of novel pesticides. Erucamide, behenic acid, palmitic acid, phenylacetic acid, and β-sitosterol have been isolated from *B. megaterium* L2 for the first time. The compounds with antibacterial activity such as phenylacetic acid, behenic acid, and β-sitosterol can be considered as novel antibacterial agents.

## Data Availability Statement

The original contributions presented in the study are included in the article/Supplementary Material, further inquiries can be directed to the corresponding author/s.

## Author Contributions

YX and QP participated in the design and experiments, data acquisition and analysis, and drafted and revised the manuscript. YJ, LY, and AX designed the experiments and contributed to data acquisition, and helped to draft the manuscript. ZL conceived the idea, participated in the design, contributed to data analysis and interpretation, and helped to revise the manuscript critically. SM and TH contributed to interpretation of the date and helped to revise the manuscript critically. YX contributed to data analysis and the determination of compounds, and helped to revise the manuscript. JZ and QZ contributed to data analysis, provided software, and helped to draft the manuscript. All authors read and approved the final manuscript.

## Conflict of Interest

The authors declare that the research was conducted in the absence of any commercial or financial relationships that could be construed as a potential conflict of interest.

## References

[B1] Akram W. Anjum T. Ali B. (2016). Phenylacetic acid is ISR determinant produced by *Bacillus fortis* IAGS162, which involves extensive re-modulation in metabolomics of tomato to protect against fusarium wilt. *Front. Plant. Sci*. 7:498. 10.3389/fpls.2016.00498 27148321 PMC4835451

[B2] Al-Thubiani A. Maher Y. A. Fathi A. Abourehab M. Alarjah M. Khan M. (2018). Identification and characterization of a novel antimicrobial peptide compound produced by *Bacillus megaterium* strain isolated from oral microflora. *Saudi. Pharm. J.* 26 1089–1097. 10.1016/j.jsps.2018.05.019 30532629 PMC6260495

[B3] Aysan Y. Sahin F. (2003). An outbreak of crown gall disease on rose caused by *Agrobacterium tumefaciens* in Turkey. *Plant. Pathol.* 52 780–780.

[B4] Bartz F. E. Glassbrook N. J. Danehower D. A. Cubeta M. A. (2013). Modulation of the phenylacetic acid metabolic complex by quinic acid alters the disease-causing activity of Rhizoctonia solani on tomato. *Phytochemistry.* 89 47–52. 10.1016/j.phytochem.2012.09.018 23380633

[B5] Biedendieck R. Borgmeier C. Bunk B. Stammen S. Scherling C. Meinhardt F. (2011). Systems biology of recombinant protein production using *Bacillus megaterium*. *Methods Enzymol.* 500 165–195. 10.1016/B978-0-12-385118-5.00010-4 21943898

[B6] Boostani H. R. Chorom M. Moezzi A. A. Enayatizamir N. (2014). Mechanisms of plant growth promoting rhizobacteria (PGPR) and mycorrhizae fungi to enhancement of plant growth under salinity stress: a review. *Sci. J. Biol. Sci.* 3 98–107.

[B7] Cater N. B. Denke M. A. (2001). Behenic acid is a cholesterol-raising saturated fatty acid in humans. *Am. J. Clin. Nutr.* 73 41–44. 10.1093/ajcn/73.1.41 11124748

[B8] Chakraborty U. Chakraborty B. Basnet M. (2006). Plant growth promotion and induction of resistance in Camellia sinensis by *Bacillus megaterium*. *J. Basic. Microbiol.* 46 186–195. 10.1002/jobm.200510050 16721878

[B9] Conner A. Dommisse E. (1992). Monocotyledonous plants as hosts for *Agrobacterium*. *Int. J. Plant. Sci.* 153 550–555. 10.2307/2995577

[B10] Cravatt B. F. Prospero-Garcia O. Siuzdak G. Gilula N. B. Henriksen S. J. Boger D. L. (1995). Chemical characterization of a family of brain lipids that induce sleep. *Science (New York, NY).* 268 1506–1509. 10.1126/science.7770779 7770779

[B11] Dhaka N. Kothari S. (2002). Phenylacetic acid improves bud elongation and in vitro plant regeneration efficiency in *Helianthus annuus L*. *Plant. Cell. Rep.* 21 29–34. 10.1007/s00299-002-0471-y 30754761

[B12] Ding C. W. Feng Q. Li C. H. (2020). Isolation and identification of antifungal components synthesized by *Bacillus megaterium* LB01 from special environment and its action mechanism. *Food. Sci.* 41 75–82.

[B13] Ding P. Qiu J. Y. Ying G. Dai L. (2014). Chemical constituents of *Millettia speciosa*. *Chin. Herb. Med.* 6 332–334.

[B14] Elmerich C. Aubert J. P. (1971). Synthesis of glutamate by a glutamine: 2-oxo-glutarate amidotransferase (NADP oxidoreductase) in *Bacillus megaterium*. *Biochem. Biophys. Res. Commun.* 42 371–376. 10.1016/0006-291x(71)90380-9 4993401

[B15] Elphinstone J. G. (2005). “The current bacterial wilt situation: a global overview,” in *Bacterial Wilt the Disease & the Ralstonia Solanacearum Species Complex*, eds Allen C. Prior P. Hayward A. C. (Minnesota, MN: APS Press), 9–28.

[B16] Eppinger M. Bunk B. Johns M. A. Edirisinghe J. N. Kutumbaka K. K. Koenig S. S. (2011). Genome sequences of the biotechnologically important *Bacillus megaterium* strains QM B1551 and DSM319. *J. Bacteriol.* 193 4199–4213. 10.1128/JB.00449-11 21705586 PMC3147683

[B17] Fernández-Ortuño D. Grabke A. Li X. Schnabel G. (2015). Independent emergence of resistance to seven chemical classes of fungicides in botrytis cinerea. *Phytopathology* 105 424–432. 10.1094/PHYTO-06-14-0161-R 25317841

[B18] Fira D. Dimkić I. Berić T. Lozo J. Stanković S. (2018). Biological control of plant pathogens by *Bacillus* species. *J. Biotechnol.* 285 44–55. 10.1016/j.jbiotec.2018.07.044 30172784

[B19] Gogoi D. Pal A. Chattopadhyay P. Paul S. Deka R. C. Mukherjee A. K. (2018). First report of plant-derived β-Sitosterol with antithrombotic, in vivo anticoagulant, and thrombus-preventing activities in a mouse model. *J. Nat. Prod.* 81 2521–2530. 10.1021/acs.jnatprod.8b00574 30406661

[B20] Goulson D. (2014). Ecology: pesticides linked to bird declines. *Nature*. 511 295–296. 10.1038/nature13642 25030159

[B21] Grace O. M. Light M. E. Lindsey K. L. Mulholland D. A. Van Staden J. Jager A. K. (2002). Antibacterial activity and isolation of active compounds from fruit of the traditional african medicinal tree kigelia africana. *S. Afr. J. Bot.* 68 220–222. 10.1016/S0254-6299(15)30424-5

[B22] Grage K. McDermott P. Rehm B. (2017). Engineering *Bacillus megaterium* for production of functional intracellular materials. *Microb. Cell. Fact.* 16:211. 10.1186/s12934-017-0823-5 29166918 PMC5700737

[B23] Guo J. Erskine P. Coker A. R. Wood S. P. Cooper J. B. (2017). Structural studies of domain movement in active-site mutants of porphobilinogen deaminase from *Bacillus megaterium*. *Acta. Crystallogr. F. Struct. Biol. Commun.* 73 612–620. 10.1107/S2053230X17015436 29095155 PMC5683031

[B24] Gupta M. Nath R. Srivastava N. Shanker K. Kishor K. Bhargava K. (1980). Anti-inflammatory and antipyretic activities of β-sitosterol. *Plant. Med.* 39 157–163. 10.1055/s-2008-1074919 6967611

[B25] Gupta S. Gupta R. Sharma S. (2013). Impact of chemical- and bio-pesticides on bacterial diversity in rhizosphere of Vigna radiata. *Ecotoxicology* 22 1479–1489. 10.1007/s10646-013-1134-1 24085606

[B26] Hamberger A. Stenhagen G. (2003). Erucamide as a modulator of water balance: new function of a fatty acid amide. *Neurochem. Res.* 28 177–185. 10.1023/a:1022364830421 12608692

[B27] Hillenga D. J. Versantvoort H. J. M. Molen S. V. D. Driessen A. J. M. Konings W. N. (1995). Penicillium chrysogenum takes up the penicillin g precursor phenylacetic acid by passive diffusion. *Appl. Environ. Microbiol.* 61 2589–2595. 10.1128/AEM.61.7.2589-2595.1995 16535072 PMC1388490

[B28] Hitchins A. D. Kahn A. J. Slepecky R. A. (1968). Interference contrast and phase contrast microscopy of sporulation and germination of Bacillus megaterium. *J. Bacteriol.* 96 1811–1817. 10.1128/JB.96.5.1811-1817.1968 4973131 PMC315245

[B29] Hu X. Roberts D. P. Xie L. Maul J. E. Yu C. Li Y. (2013). *Bacillus megaterium* A6 suppresses sclerotinia sclerotiorum on oilseed rape in the field and promotes oilseed rape growth. *Crop Prot.* 52 151–158. 10.1016/j.cropro.2013.05.018

[B30] Husain S. Jain A. Kothari S. L. (1999). Phenylacetic acid improves bud elongation and in vitro plant regeneration efficiency in *Capsicum annuum L*. *Plant Cell Rep.* 19 64–68. 10.1007/s002990050711 30754761

[B31] Hwang B. K. Lim S. W. Kim B. S. Lee J. Y. Moon S. S. (2001). Isolation and in vivo and in vitro antifungal activity of phenylacetic acid and sodium phenylacetate from *Streptomyces humidus*. *Appl. Environ. Microbiol.* 67 3739–3745. 10.1128/AEM.67.8.3739-3745.2001 11472958 PMC93082

[B32] Ji Y. Y. Dai Y. F. Chen X. Li Z. Xiao Y. Yang L. (2019). Analysis of antibacterial effect and components of crude extract from *Bacillus megaterium* L2. *China Brew.* 38 120–124.

[B33] Ji Y. Y. Huang H. Xiao Y. Chen X. Dai Y. F. Li Z. (2018). Optimization of fermentation conditions of antagonistic bacterium *Bacillus megaterium* L2 by response surface methodology. *China Brew.* 37 107–112.

[B34] Joy Hoskeri H. Krishna V. Jignesh S. Sanjay S. T. Roshan A. Vijay S. (2012). In-silico drug designin using b-sitosterol isolated from flaveria trinervia against peptide deformylase protein tohypothesize bactericidal effect. *Int. J. Pharm. Pharm. Sci.* 4 192–196.

[B35] Kamal N. Liu Z. Qian C. Wu J. Zhong X. (2021). Improving hybrid Pennisetum growth and cadmium phytoremediation potential by using *Bacillus megaterium* BM18-2 spores as biofertilizer. *Microbiol. Res.* 242:126594. 10.1016/j.micres.2020.126594 33007635

[B36] Kawazu K. Zhang H. Yamashita H. Kanzaki H. (1996). Relationship between the pathogenicity of the pine wood nematode, Bursaphelenchus xylophilus, and phenylacetic acid production. *Biosci. Biotechnol. Biochem.* 60 1413–1415. 10.1271/bbb.60.1413 8987588

[B37] Kayser O. Kolodziej H. (1997). Antibacterial activity of extracts and constituents of Pelargonium sidoides and Pelargonium reniforme. *Planta Med.* 63 508–510. 10.1055/s-2006-957752 9434601

[B38] Kim C. R. Kim H. S. Choi S. J. Kim J. K. Gim M. C. Kim Y. J. (2018). Erucamide from radish leaves has an inhibitory effect against acetylcholinesterase and prevents memory deficit induced by trimethyltin. *J. Med. Food.* 21 769–776. 10.1089/jmf.2017.4117 30110203

[B39] Kim Y. Cho J. Y. Kuk J. H. Moon J. H. Cho J. I. Kim Y. C. (2004). Identification and antimicrobial activity of phenylacetic acid produced by Bacillus licheniformis isolated from fermented soybean, Chungkook-Jang. *Curr. Microbiol.* 48 312–317. 10.1007/s00284-003-4193-3 15057459

[B40] Lammers M. Nahrstedt H. Meinhardt F. (2004). The *Bacillus megaterium* comE locus encodes a functional DNA uptake protein. *J. Basic Microbiol.* 44 451–458. 10.1002/jobm.200410450 15558816

[B41] Lee M. H. Lee J. Nam Y. D. Lee J. S. Seo M. J. Yi S. H. (2016). Characterization of antimicrobial lipopeptides produced by *Bacillus* sp. LM7 isolated from c*hungkookjang*, a Korean traditional fermented soybean food. *Int. J. Food Microbiol.* 221 12–18. 10.1016/j.ijfoodmicro.2015.12.010 26803269

[B42] Li L. Luo D. Y. Liu Y. (2001). Studies on antibiotic active constituents of *Balaps japanensis yunnanensis*. *Chin. Tradit. Herbal. Drugs.* 3 7–9.

[B43] Li M. M. Jiang Z. E. Song L. Y. Quan Z. S. Yu H. L. (2017). Antidepressant and anxiolytic-like behavioral effects of erucamide, a bioactive fatty acid amide, involving the hypothalamus-pituitary-adrenal axis in mice. *Neurosci. Lett.* 640 6–12. 10.1016/j.neulet.2016.12.072 28082151

[B44] Li W. H. Chang S. T. Chang S. C. Chang H. T. (2008). Isolation of antibacterial diterpenoids from Cryptomeria japonica bark. *Nat. Prod. Res.* 22 1085–1093. 10.1080/14786410802267510 18780250

[B45] Lu Y. F. Zhou Y. R. Nakai S. Hosomi M. Zhang H. Kronzucker H. J. (2014). Stimulation of nitrogen removal in the rhizosphere of aquatic duckweed by root exudate components. *Planta.* 239 591–603. 10.1007/s00425-013-1998-6 24271005 PMC3928532

[B46] Malten M. Hollmann R. Deckwer W. D. Jahn D. (2005). Production and secretion of recombinant Leuconostoc mesenteroides dextransucrase DsrS in *Bacillus megaterium*. *Biotechnol. Bioeng.* 89 206–218. 10.1002/bit.20341 15593264

[B47] Mannaa M. Kim K. D. (2018). Biocontrol activity of volatile-producing *Bacillus megaterium* and *Pseudomonas protegens* against *Aspergillus* and *Penicillium* spp. predominant in stored rice grains: study II. *Mycobiology* 46 52–63. 10.1080/12298093.2018.1454015 29998033 PMC6037079

[B48] Munoz-Leoz B. Garbisu C. Charcosset J. Y. Sanchez-Perez J. M. Antiguedad I. Ruiz-Romera E. (2013). Non-target effects of three formulated pesticides on microbially-mediated processes in a clay-loam soil. *Sci. Total Environ.* 449 345–354. 10.1016/j.scitotenv.2013.01.079 23454695

[B49] Nascimento F. X. Hernández A. G. Glick B. R. Rossi M. J. (2019). Plant growth-promoting activities and genomic analysis of the stress-resistant *Bacillus megaterium* STB1, a bacterium of agricultural and biotechnological interest. *Biotechnol. Rep (Amst).* 25:e00406. 10.1016/j.btre.2019.e00406 31886139 PMC6920507

[B50] Nicolopoulou-Stamati P. Maipas S. Kotampasi C. Stamatis P. Hens L. (2016). Chemical pesticides and human health: the urgent need for a new concept in agriculture. *Front. Public. Health* 4:148. 10.3389/fpubh.2016.00148 27486573 PMC4947579

[B51] Nishanth Kumar S. Mohandas C. Siji J. V. Rajasekharan K. N. Nambisan B. (2012). Identification of antimicrobial compound, diketopiperazines, from a *Bacillus* sp. N strain associated with a rhabditid entomopathogenic nematode against major plant pathogenic fungi. *J. Appl. Microbiol.* 113 914–924. 10.1111/j.1365-2672.2012.05385.x 22747978

[B52] Pi K. (2009). *Study on Chemical Constituents and Biological Activity of TEUCRIUM Labiosum.* Ph.D. dissertation, University of Guizhou, Guiyang.

[B53] Płaza G. Turek A. Król E. Szczygłowska R. (2013). Antifungal and antibacterial properties of surfactin isolated from *Bacillus subtilis* growing on molasses. *Afr. J. Microbiol. Res.* 7 3165–3170. 10.5897/AJMR2013.5565

[B54] Qin J. (2013). *Optimization of Fermentation Conditions and Purification of the Antifungal Substance Produced by Bacillus megaterium* 196. Ph. D. dissertation, University of Guangxi, Nanning.

[B55] Quigley E. M. (2010). Prebiotics and probiotics; modifying and mining the microbiota. *Pharmacol. Res.* 61 213–218. 10.1016/j.phrs.2010.01.004 20080184

[B56] Rahman M. M. Ali M. E. Khan A. A. Akanda A. M. Uddin M. K. Hashim U. (2012). Isolation, characterization, and identification of biological control agent for potato soft rot in Bangladesh. *Sci. World J.* 2012:723293. 10.1100/2012/723293 22645446 PMC3356727

[B57] Ryan P. R. Dessaux Y. Thomashow L. S. Weller D. M. (2009). Rhizosphere engineering and management for sustainable agriculture. *Plant Soil* 321 363–383.

[B58] Sen A. Dhavan P. Shukla K. K. Singh S. Tejovathi G. (2012). Analysis of IR, NMR and antimicrobial activity of β-sitosterol isolated from *momordica charantia*. *Sci. Secure J. Biotech.* 1 9–13.

[B59] Slininger P. J. Burkhead K. D. Schisler D. A. (2004). Antifungal and sprout regulatory bioactivities of phenylacetic acid, indole-3-acetic acid, and tyrosol isolated from the potato dry rot suppressive bacterium *Enterobacter cloacae* S11:T:07. *J. Ind. Microbiol. Biotechnol.* 31 517–524. 10.1007/s10295-004-0180-3 15558349

[B60] Sopheareth M. Chan S. Naing K. W. Lee Y. S. Hyun H. N. Kim Y. C. (2013). Biocontrol of late blight (Phytophthora capsici) disease and growth promotion of pepper by Burkholderia cepacia MPC-7. *Plant. Pathol. J.* 29 67–76. 10.5423/PPJ.OA.07.2012.0114 25288930 PMC4174795

[B61] Sun L. Lu Y. F. Kronzucker H. J. Shi W. M. (2016). Quantification and enzyme targets of fatty acid amides from duckweed root exudates involved in the stimulation of denitrification. *J. Plant Physiol.* 198 81–88. 10.1016/j.jplph.2016.04.010 27152459

[B62] Sun T. Li M. Saleem M. Zhang X. Zhang Q. (2020). The fungicide “fluopyram” promotes pepper growth by increasing the abundance of P-solubilizing and N-fixing bacteria. *Ecotoxicol. Environ. Saf.* 188:109947. 10.1016/j.ecoenv.2019.109947 31744624

[B63] Tamilmani E. Radhakrishnan R. Sankaran K. (2018). 13-Docosenamide release by bacteria in response to glucose during growth-fluorescein quenching and clinical application. *Appl. Microbiol. Biotechnol.* 102 6673–6685. 10.1007/s00253-018-9127-x 29860593

[B64] Toth I. K. Bell K. S. Holeva M. C. Birch P. R. (2003). Soft rot erwiniae: from genes to genomes. *Mol. Plant Pathol.* 4 17–30. 10.1046/j.1364-3703.2003.00149.x 20569359

[B65] Wakamatsu K. Masaki T. Itoh F. Kondo K. Sudo K. (1990). Isolation of fatty acid amide as an angiogenic principle from bovine mesentery. *Biochem. Biophys. Res. Commun.* 168 423–429. 10.1016/0006-291x(90)92338-z 2334413

[B66] Wightman F. Lighty D. L. (1982). Identification of phenylacetic acid as a natural auxin in the shoots of higher plants. *Physiol. Plant.* 55 17–24. 10.1111/j.1399-3054.1982.tb00278.x

[B67] Wu X. Liu Y. Sheng W. Sun J. Qin G. (1997). Chemical constituents of *Isatis indigotica*. *Planta Med.* 63 55–57. 10.1055/s-2006-957604 17252328

[B68] Xie S. Yang F. Tao Y. Chen D. Qu W. Huang L. (2017). Enhanced intracellular delivery and antibacterial efficacy of enrofloxacin-loaded docosanoic acid solid lipid nanoparticles against intracellular *Salmonella*. *Sci. Rep.* 7:41104. 10.1038/srep41104 28112240 PMC5253767

[B69] Yang L. N. He M. H. Ouyang H. B. Zhu W. Pan Z. C. Sui Q. J. (2019). Cross-resistance of the pathogenic fungus Alternaria alternata to fungicides with different modes of action. *BMC Microbiol.* 19:205. 10.1186/s12866-019-1574-8 31477005 PMC6720428

[B70] Yff B. T. Lindsey K. L. Taylor M. B. Erasmus D. G. Jäger A. K. (2002). The pharmacological screening of Pentanisia prunelloides and the isolation of the antibacterial compound palmitic acid. *J. Ethnopharmacol.* 79 101–107. 10.1016/s0378-8741(01)00380-4 11744302

[B71] Zhang J. H. (2015). *Studies on Optimization of Fermentation Conditions and Antimicrobial Activive Compositions and Nematicidal Activity of the Fermentation Liquid of Trichoderma Longibrachiatum* T6. Ph.D. Dissertation, University of Gansu Agricultural, Gansu.

[B72] Zhang Q. Saleem M. Wang C. (2019). Effects of biochar on the earthworm (Eisenia foetida) in soil contaminated with and/or without pesticide mesotrione. *Sci. Total. Environ.* 671 52–58. 10.1016/j.scitotenv.2019.03.364 30927727

[B73] Zhao J. Y. Xiao Y. Yang L. Zhang S. Ji Y. Y. Li Z. (2019). Antibacterial Mechanism of Fermentation Product from *Bacillus megaterium* L2 against *Erwinia carotovora* subsp. *Carotovora*. *Food Sci.* 40 14–20. 10.7506/spkx1002-6630-20181112-124

[B74] Zhou Y. P. Wu R. Shen W. Yu H. H. Yu S. J. (2018). Comparison of effects of oleic acid and palmitic acid on lipid deposition and mTOR/S6K1/SREBP-1c pathway in HepG2 cells. *Zhonghua Gan Zang Bing Za Zhi.* 26 451–456. 10.3760/cma.j.issn.1007-3418.2018.06.012 30317760 PMC12769202

[B75] Zock P. L. de Vries J. H. Katan M. B. (1994). Impact of myristic acid versus palmitic acid on serum lipid and lipoprotein levels in healthy women and men. *Arterioscler Thromb.* 14 567–575. 10.1161/01.atv.14.4.567 8148355

