# Supplementary material for: Isolation and Identification of Antibacterial Bioactive Compounds From Bacillus megaterium L2
Source: Front Microbiol. 2021 Mar 24;12:645484. doi: 10.3389/fmicb.2021.645484 (PMC8024468; doi:10.3389/fmicb.2021.645484)
Supplement: Supplementary file 1 [file Data_Sheet_1.pdf]

**Supplementary Materials**

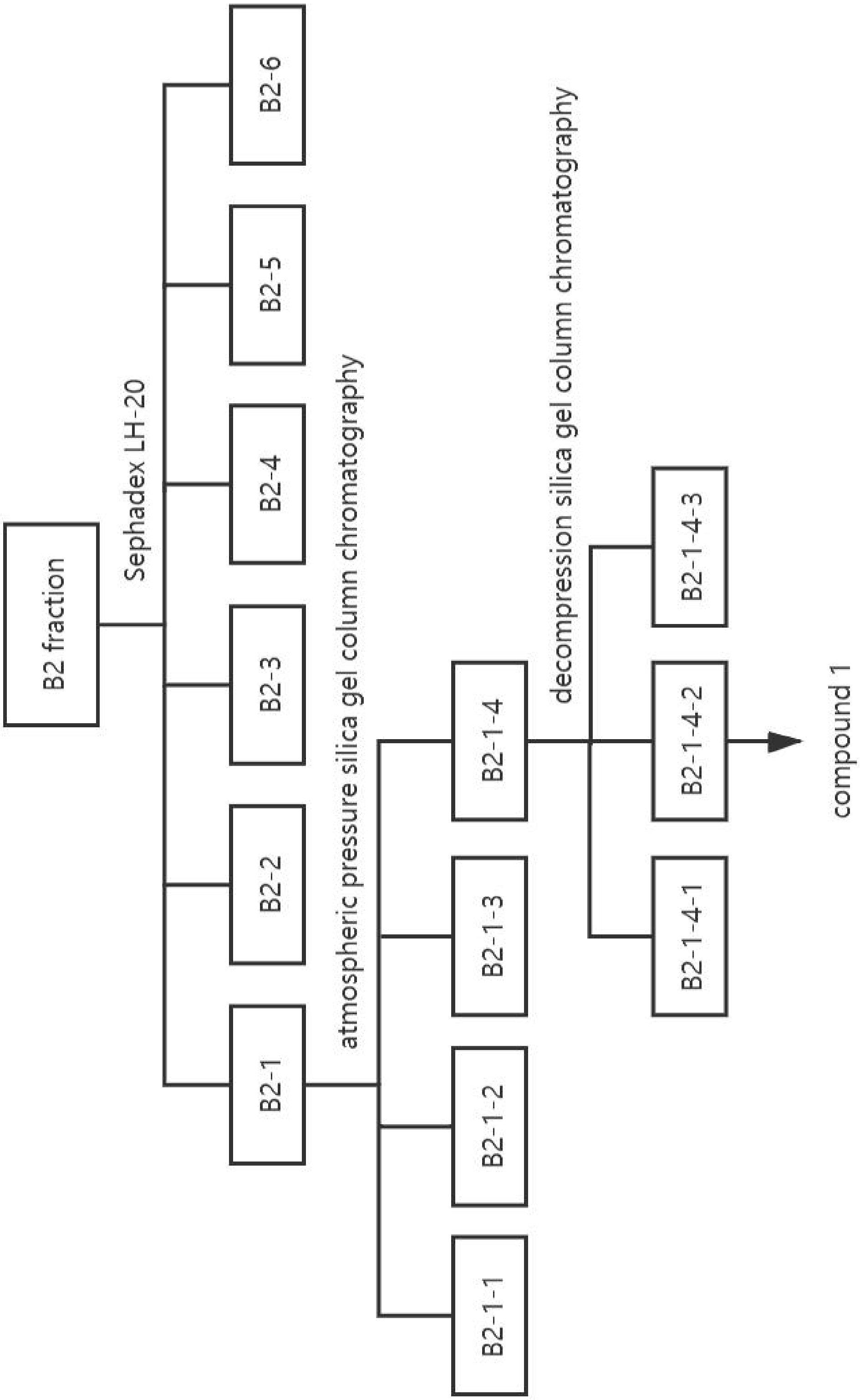


**Supplementary Figure 1**. The antibacterial activity guided fractionation of B2 fraction.

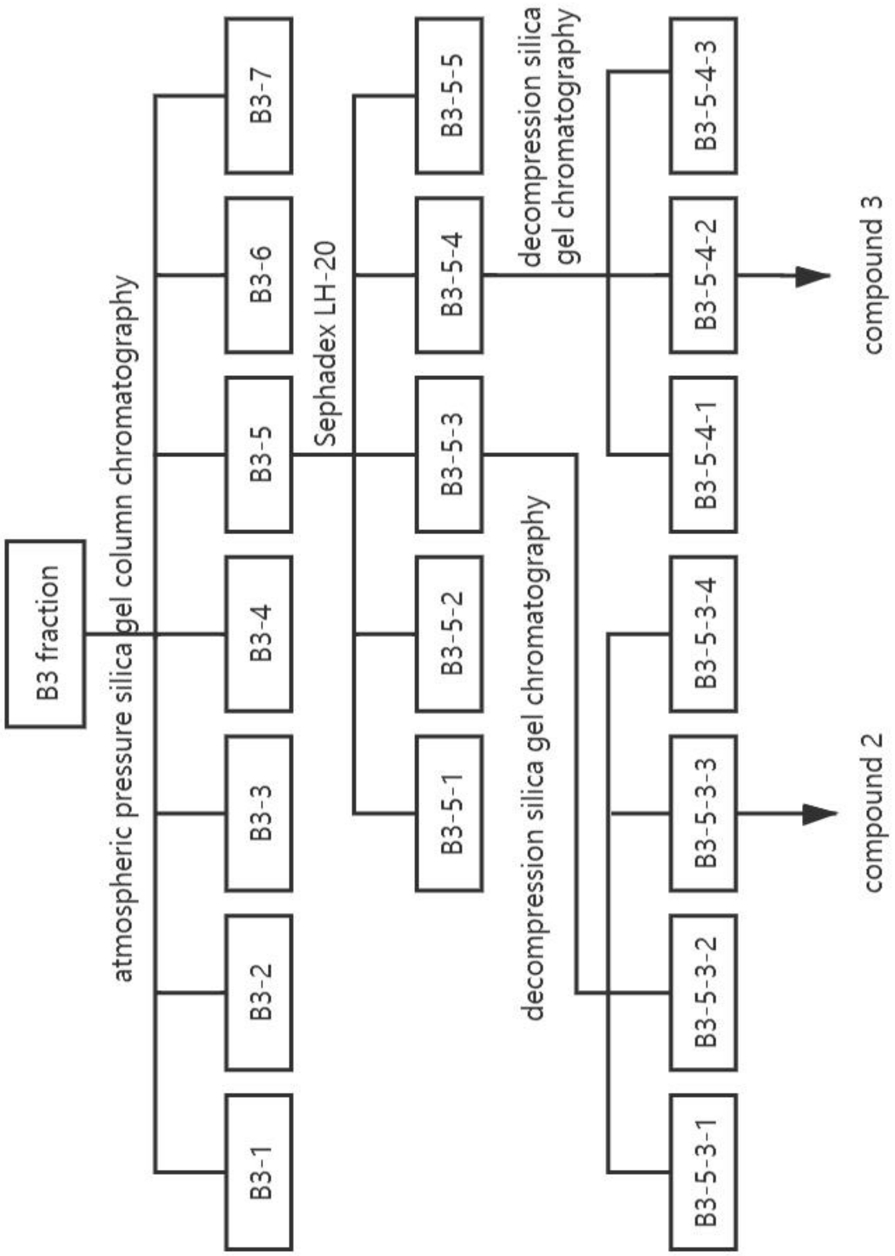


**Supplementary Figure 2**. The antibacterial activity guided fractionation of B3 fraction.

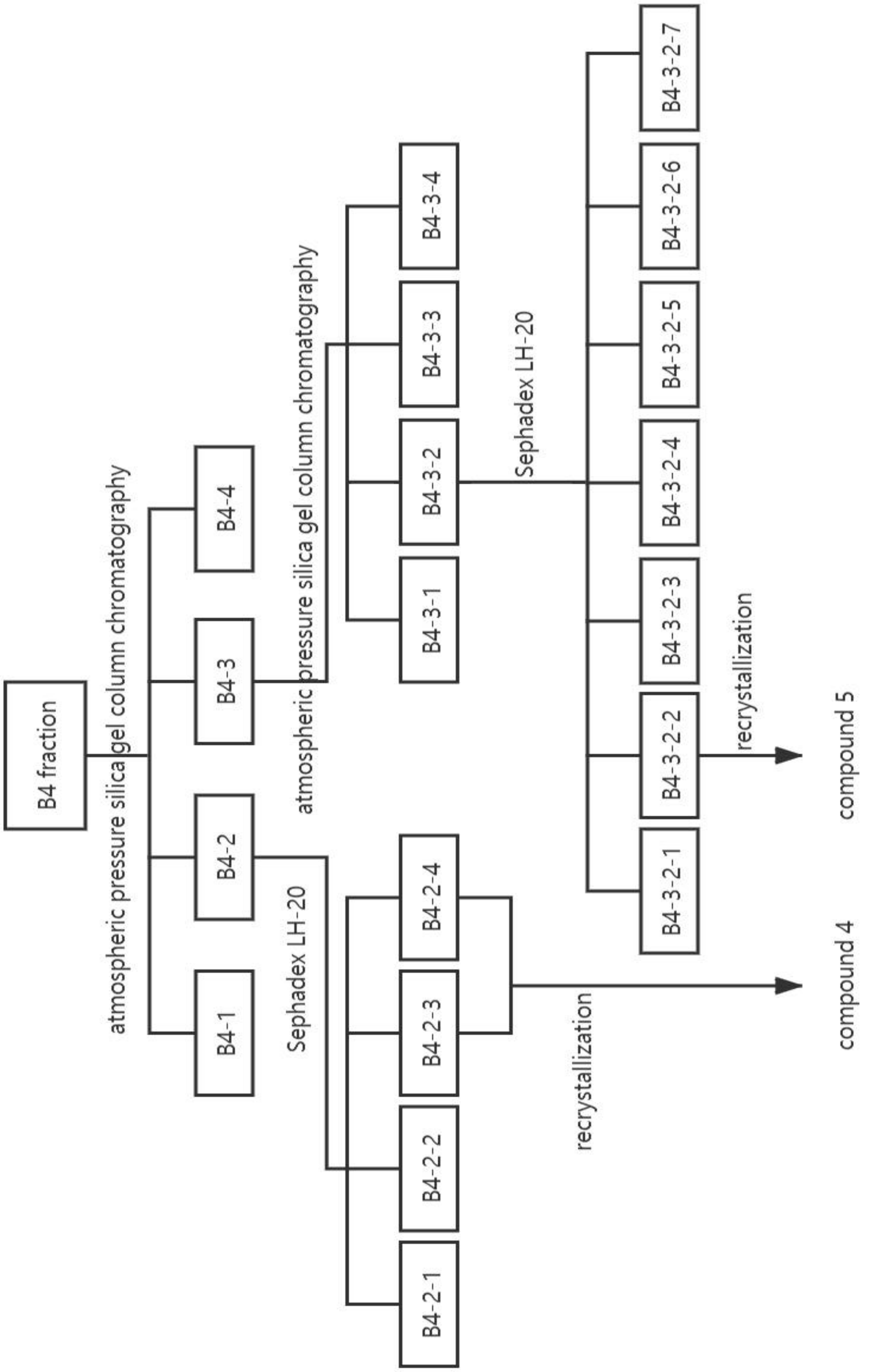


**Suppleme**
**ntary Figure 3**. The antibacterial activity guided fractionation of B4 fraction.

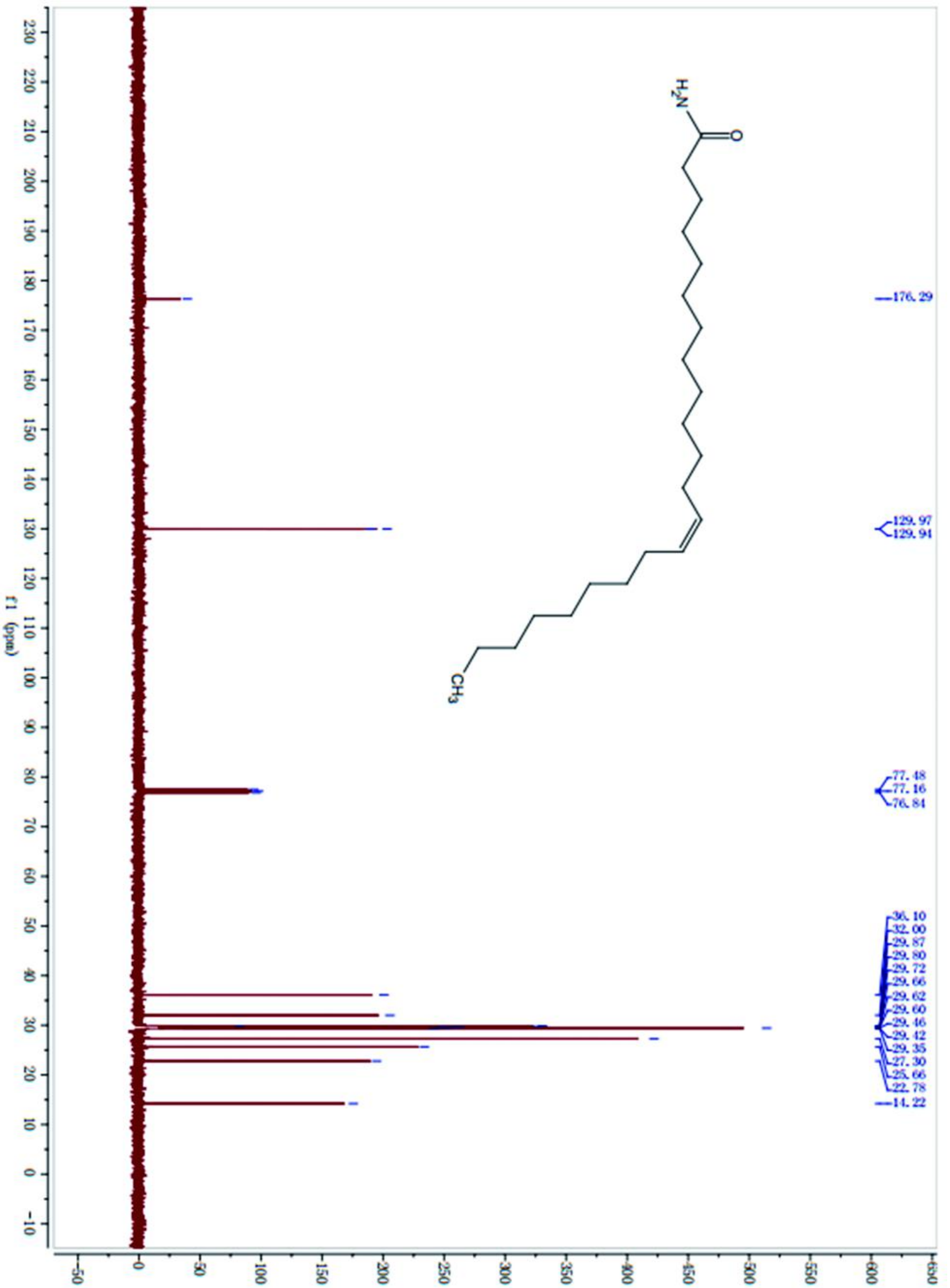


**Supplementary Figure 4.** The $^{13}$C-NMR spectrum of erucamide.

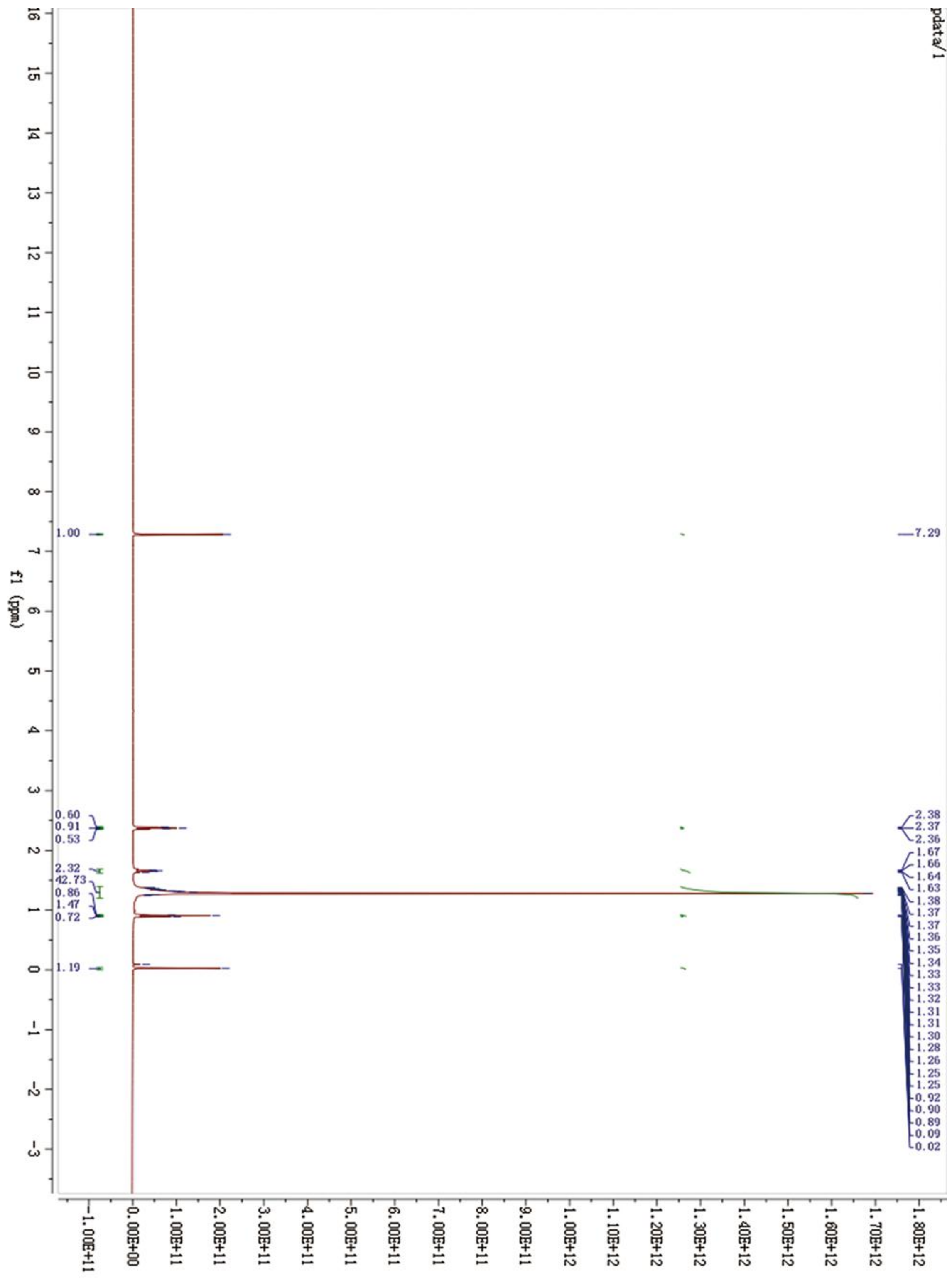


**Supplementary Figure 5.** The $^{1}$H NMR spectrum of behenic acid.

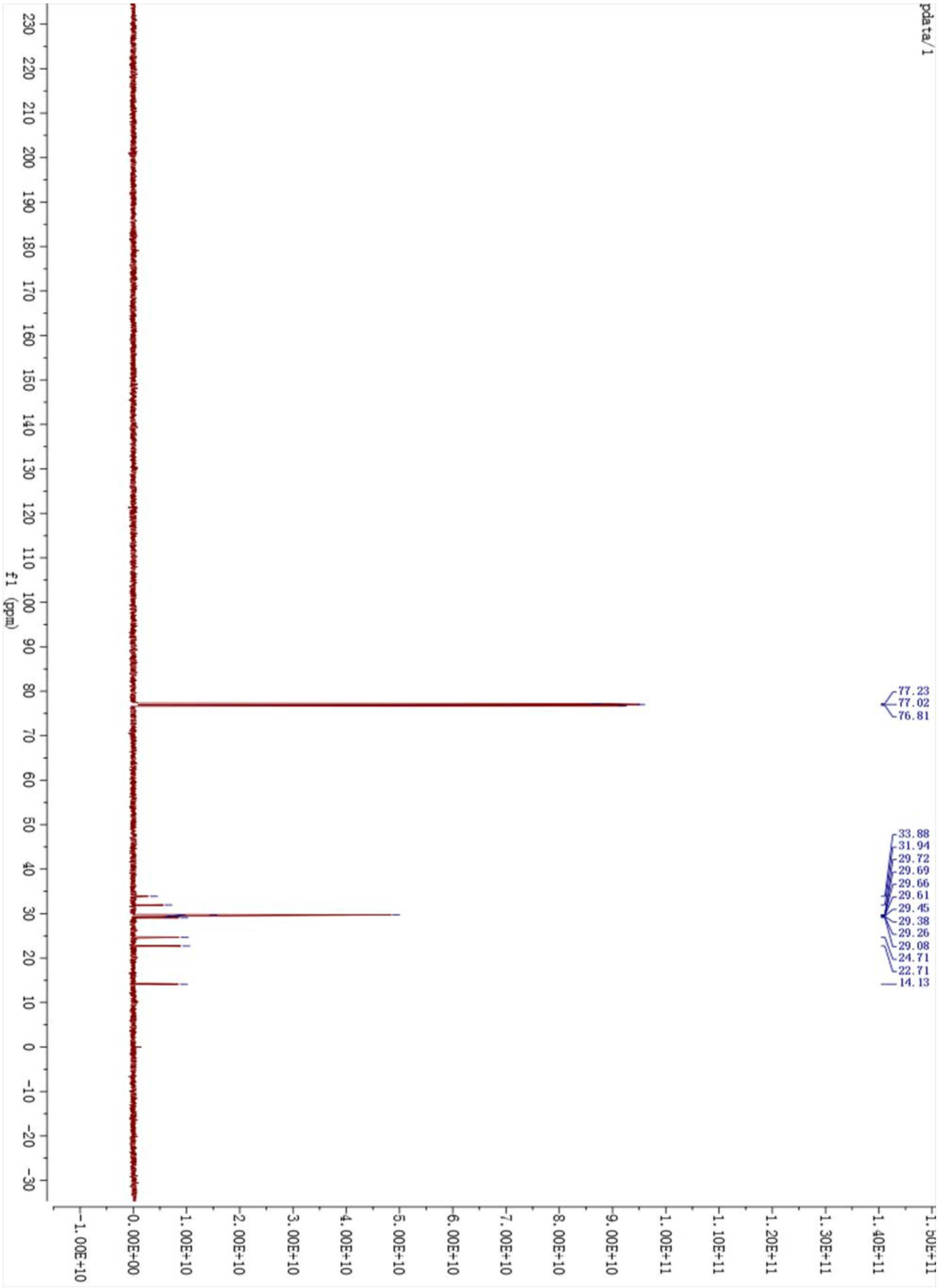


**Supplementary Figure 6.** The $^{13}C$-NMR spectrum of behenic acid.

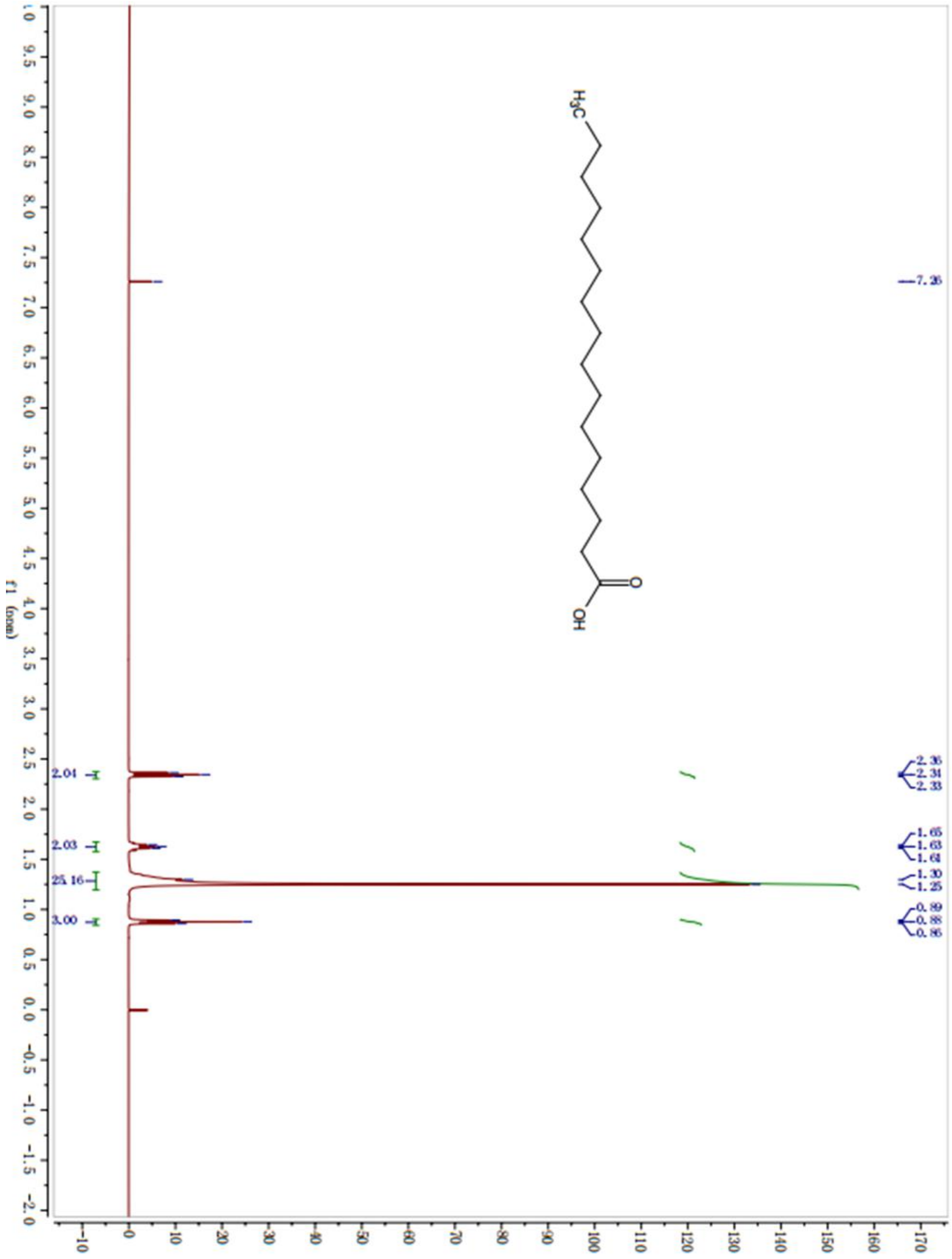


**Supplementary Figure 7.**The $^{1}$H NMR spectrum of palmitic acid.

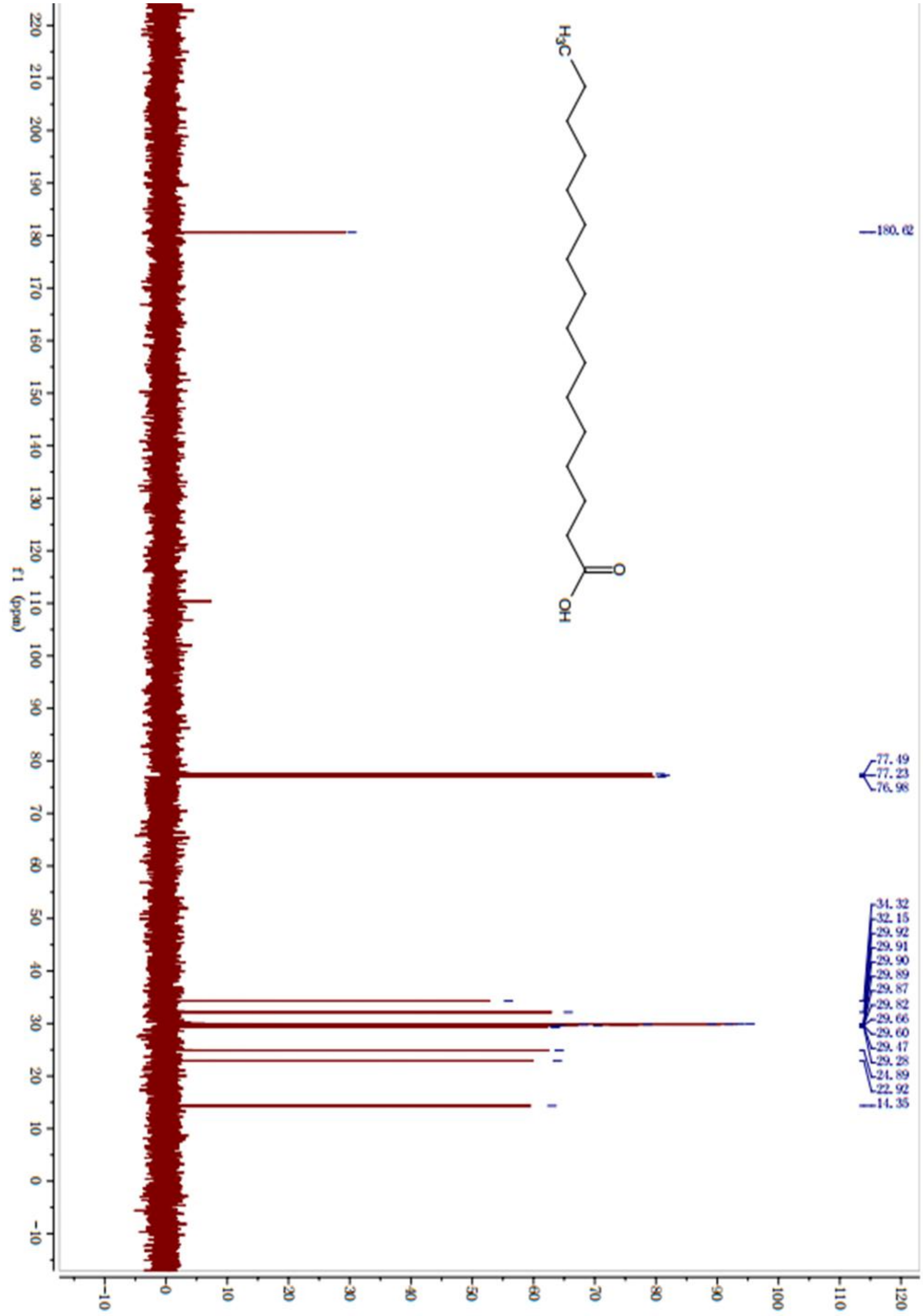


**Supplementary Figure 8.** $^{13}$C-NMR spectrum of palmitic acid.

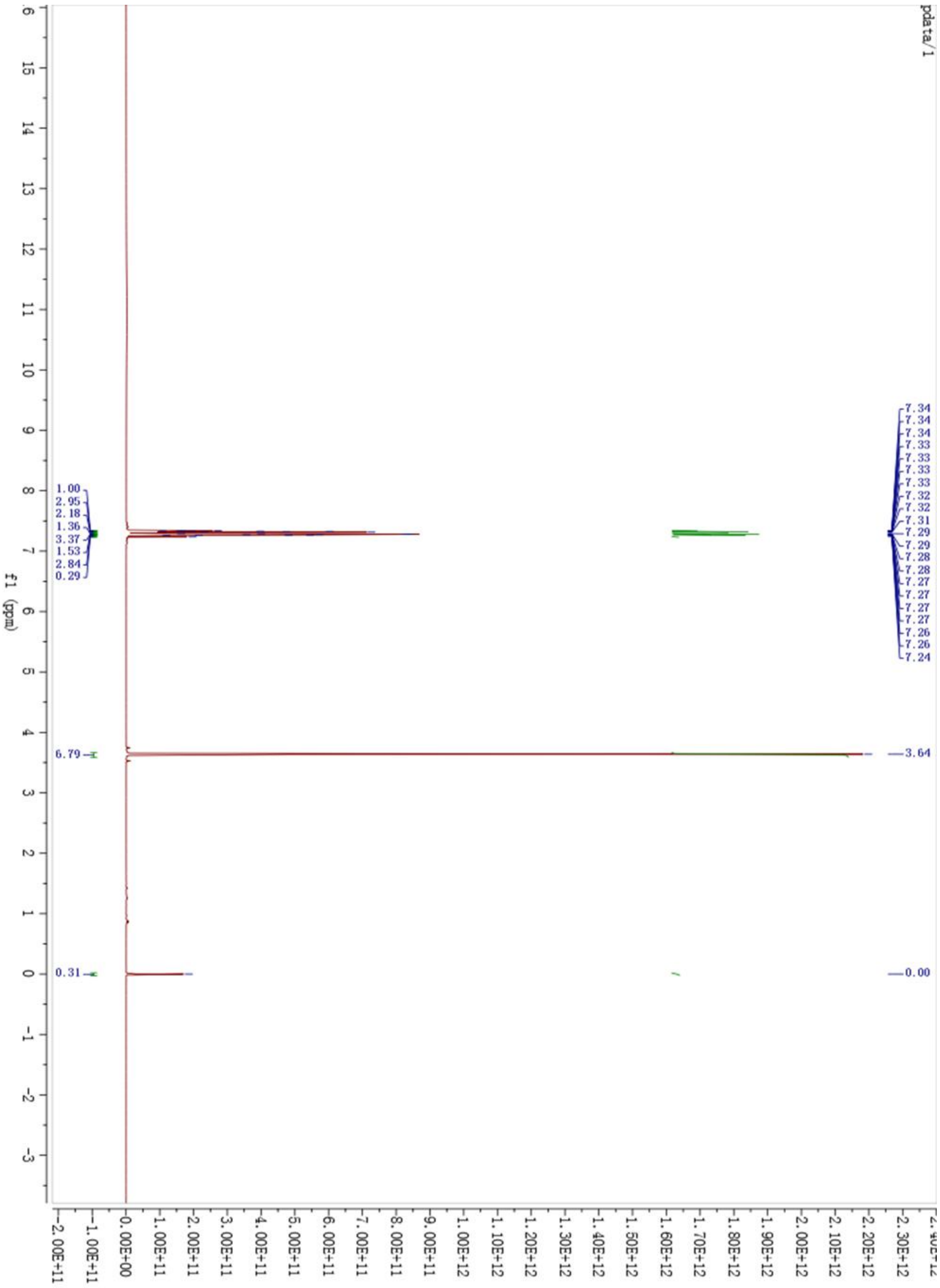


**Supplementary Figure 9.** The $^{1}H$-NMR spectrum of phenylacetic acid.

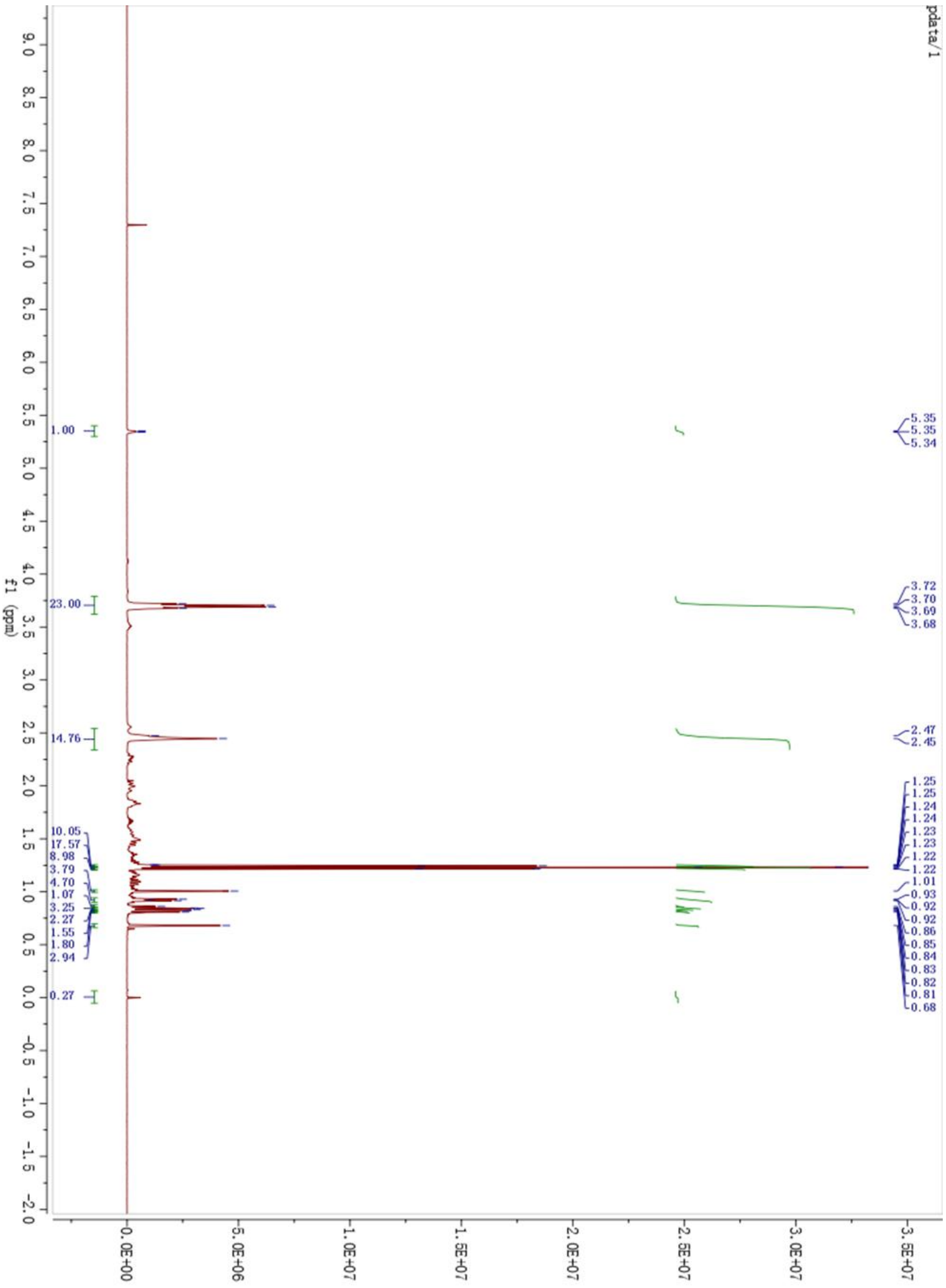


**Supplementary Figure 10.** The $^{1}$H NMR spectrum of β-sitosterol.

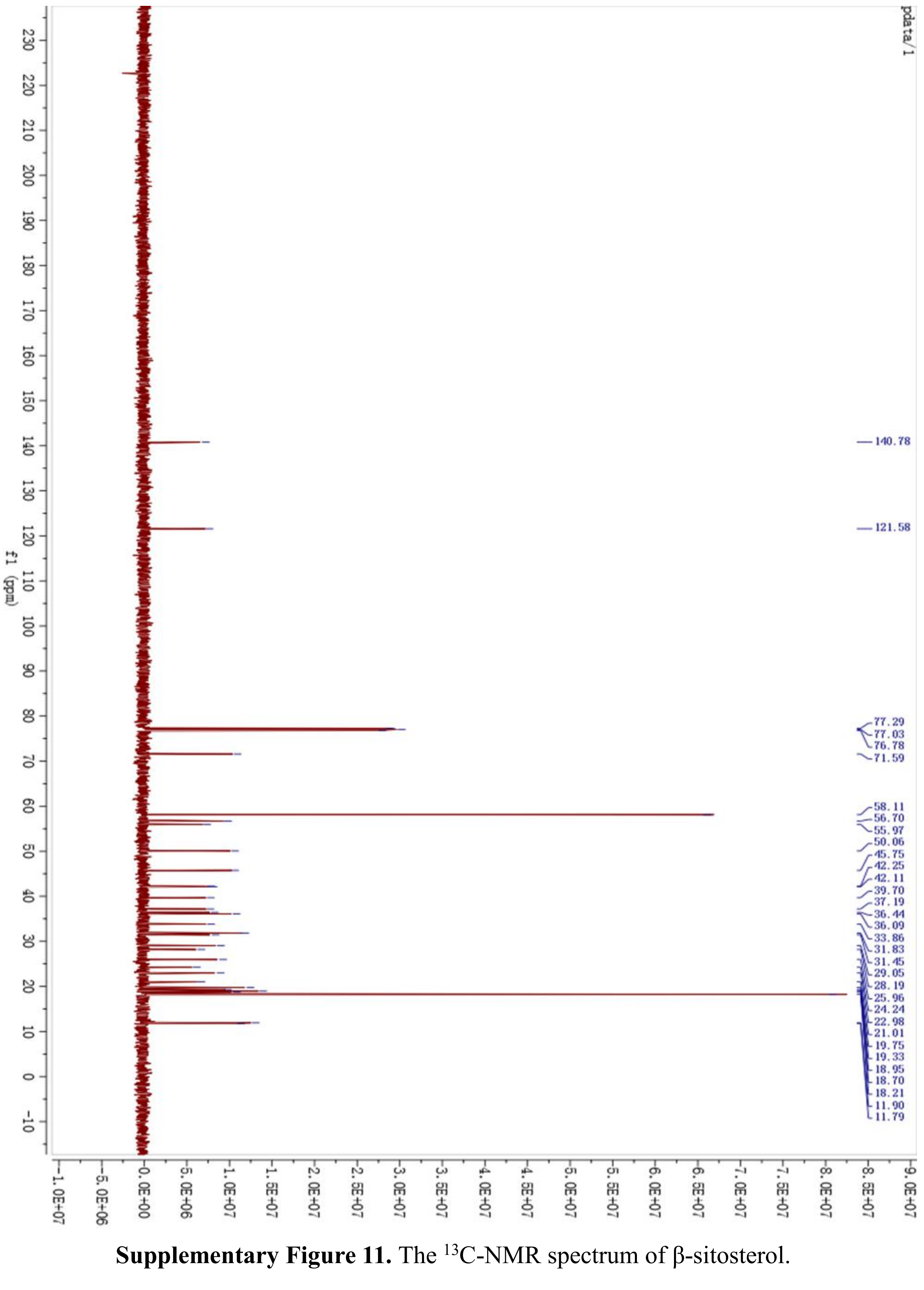

**Supplementary Figure 11.** The $^{13}$C-NMR spectrum of β-sitosterol.
